# Dynamics of Melanoma-Associated Epitope-Specific CD8+ T Cells in the Blood Correlate With Clinical Outcome Under PD-1 Blockade

**DOI:** 10.3389/fimmu.2022.906352

**Published:** 2022-07-07

**Authors:** Andrea Gaißler, Trine Sundebo Meldgaard, Christina Heeke, Sepideh Babaei, Siri Amanda Tvingsholm, Jonas Bochem, Janine Spreuer, Teresa Amaral, Nikolaus Benjamin Wagner, Reinhild Klein, Friedegund Meier, Claus Garbe, Thomas K. Eigentler, Graham Pawelec, Manfred Claassen, Benjamin Weide, Sine Reker Hadrup, Kilian Wistuba-Hamprecht

**Affiliations:** ^1^ Department of Dermatology, University Hospital Tübingen, Eberhard Karls University of Tübingen, Tübingen, Germany; ^2^ Internal Medicine I, University Hospital Tübingen, Eberhard Karls University of Tübingen, Tübingen, Germany; ^3^ Department of Health Technology, Danmarks Tekniske Universitet (DTU) HEALTH TECH, Copenhagen, Denmark; ^4^ Excellence Cluster (EXC) 2180, “Image Guided and Functionally Instructed Tumor Therapies” (iFIT), Tübingen, Germany; ^5^ Department of Dermatology, Venereology and Allergology, Kantonsspital St. Gallen, St. Gallen, Switzerland; ^6^ Internal Medicine II, University Hospital Tübingen, Eberhard Karls University of Tübingen, Tübingen, Germany; ^7^ Skin Cancer Center at the University Cancer Centre and National Center for Tumor Diseases Dresden, Dresden, Germany; ^8^ Department of Dermatology, Faculty of Medicine and University Hospital Carl Gustav Carus, Technische Universität Dresden, Dresden, Germany; ^9^ Department of Dermatology, Venereology and Allergology, Charité – Universitätsmedizin Berlin, corporate member of Freie Universität Berlin and Humboldt-Universität zu Berlin, Berlin, Germany; ^10^ Department of Immunology, Interfaculty Institute for Cell Biology, Eberhard Karls University Tübingen, Tübingen, Germany; ^11^ Health Sciences North Research Institute, Sudbury, ON, Canada; ^12^ Department of Computer Science, Eberhard Karls University of Tübingen, Tübingen, Germany

**Keywords:** T cells, checkpoint blockade, melanoma, melanoma-associated antigen, regression analysis, dextramer

## Abstract

Immune checkpoint blockade (ICB) is standard-of-care for patients with metastatic melanoma. It may re-invigorate T cells recognizing tumors, and several tumor antigens have been identified as potential targets. However, little is known about the dynamics of tumor antigen-specific T cells in the circulation, which might provide valuable information on ICB responses in a minimally invasive manner. Here, we investigated individual signatures composed of up to 167 different melanoma-associated epitope (MAE)-specific CD8+ T cells in the blood of stage IV melanoma patients before and during anti-PD-1 treatment, using a peptide-loaded multimer-based high-throughput approach. Additionally, checkpoint receptor expression patterns on T cell subsets and frequencies of myeloid-derived suppressor cells and regulatory T cells were quantified by flow cytometry. Regression analysis using the MAE-specific CD8+ T cell populations was applied to identify those that correlated with overall survival (OS). The abundance of MAE-specific CD8+ T cell populations, as well as their dynamics under therapy, varied between patients. Those with a dominant increase of these T cell populations during PD-1 ICB had a longer OS and progression-free survival than those with decreasing or balanced signatures. Patients with a dominantly increased MAE-specific CD8+ T cell signature also exhibited an increase in TIM-3+ and LAG-3+ T cells. From these results, we created a model predicting improved/reduced OS by combining data on dynamics of the three most informative MAE-specific CD8+ T cell populations. Our results provide insights into the dynamics of circulating MAE-specific CD8+ T cell populations during ICB, and should contribute to a better understanding of biomarkers of response and anti-cancer mechanisms.

## Introduction

Immune checkpoint blockade (ICB) (1, 2) has revolutionized the treatment of metastatic melanoma and of an increasing number of other solid cancers (3, 4). Monotherapy with antagonistic antibodies targeting programmed cell death receptor 1 (PD-1, CD279) on the surface of T cells or combination therapy with antagonistic antibodies against cytotoxic T-lymphocyte-associated protein-4 (CTLA-4, CD152) are now standard-of-care treatments for patients with advanced melanoma with 5-year survival rates of approximately 50% (5). Thus, unfortunately, not all patients experience durable clinical benefit (6). Hence, a better understanding of the modes of action of ICB, and biomarkers predicting clinical outcome, are urgently required. Candidate markers such as frequencies of myeloid-derived suppressor cells (MDSCs), or T cell populations with certain phenotypes possess promising biomarker characteristics (7–12). However, defining tumor-specificity of T cells based on their phenotype is challenging, and the described cellular populations often lack proven specificity for the tumor. The high mutational burden of melanoma (13, 14) results in a potentially large number of T cell neo-epitopes derived specifically from the cancer mutagenome and is thus thought to be a key driver of successful ICB (15, 16). T cells recognizing such neo-epitopes may play an important role in anti-tumor immunity by recognizing cancer mutations unique to the tumor and eliminating the cells carrying them (17, 18). Antigens derived from non-mutated genes which are abnormally expressed, or expressed only at low levels by normal tissues [i.e. shared tumor-associated antigens (TAA)], may also contribute to tumor elimination through T cell recognition (19). Consequently, the identification of the products of such abnormally expressed genes that are also immunogenic is a promising approach for the development of new immunotherapy concepts. Unfortunately, due to inter- and intratumor heterogeneity, especially in a metastatic setting, this is only possible in a fragmented manner. An elegant alternative to study such tumor antigens expressed by primary tumors or metastases is to investigate tumor-specific T cells in the peripheral blood, which is the compartment allowing for cellular exchange between different tissues as well as metastases.

Using tests of T cell function, in metastatic melanoma patients not receiving anti-PD-1 treatment we have previously shown that the presence of circulating NY-ESO-1 and Melan-A-reactive T-cells was associated with prolonged overall survival (OS) (7, 20, 21). Also, earlier studies by others revealed that these TAAs might be promising targets for active interventions (22–24). Recently, we reported that the dynamics of NY-ESO-1- and Melan-A-reactive T cells under PD-1 ICB are associated with clinical outcome (25). However, data on the impact of ICB on the presence of circulating TAA-specific T cells (beyond established epitopes like those from NY-ESO-1 or Melan-A) and conclusively the presence of their antigens in the tumors is still limited. However, this is urgently required to supplement the range of T cell targets potentially recognized as a result of ICB induced/modulated TAA expression patterns in the tumor. This could lead to a significantly better understanding of ICB-associated changes/accessibility of TAAs in the tumors of patients who have been successfully treated, and thereby provide opportunities for the development of new immunotherapeutic treatments. Thus, the aim of the present study was to characterize a broad spectrum of a large number of different melanoma-associated epitope (MAE)-specific CD8+ T cell populations and their dynamics in the peripheral blood of anti-PD-1-treated patients to screen for further TAAs with potential clinical relevance. Therefore, we used a defined panel of 167 major histocompatibility complex (MHC) MAE dextramers in an *ex vivo*, high-throughput analytical approach, that also allows the detection of low affinity as well as high affinity T cell receptors (TCR) (26).

## Material and Methods

### Patient Material

**Figure 1:**
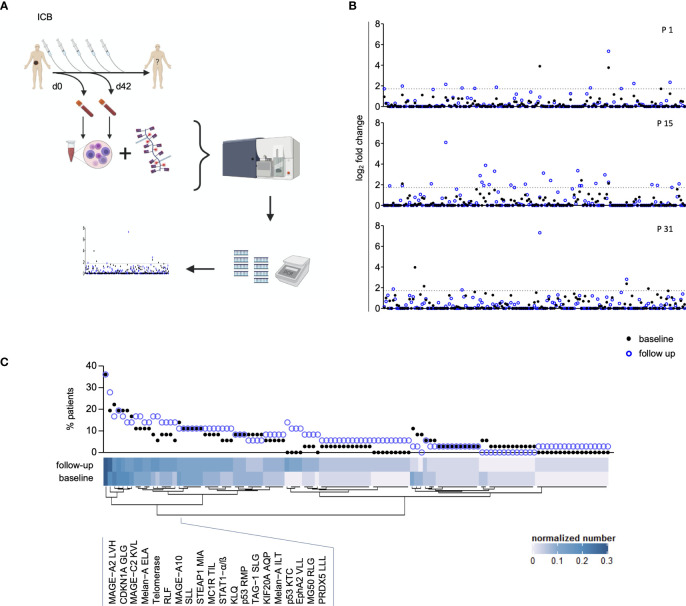
Assessment of peripheral blood Melanoma-Associated Epitope (MAE)-specific CD8+ T cell signatures. The study design is depicted in **(A)** and was created with BioRender.com. Peripheral blood T cell screening profiles of three representative patients using 167 melanoma-associated epitopes. Data on the y-axis present the detected log fold-change of the individual T cell population (x-axis) relative to the input sample. The dotted lines represent the selected threshold level log_2_FC **(B)**. The relative abundance of all detected 117 MAE-specific CD8+ T cells at baseline and follow-up within the investigated cohort is depicted in the scatter plot. The normalized numbers of each specific T cell population are illustrated in the heat map **(C)**.

Venous blood samples were obtained from HLA-A*0201+ stage IV melanoma patients (n=36) before [baseline (BL)] and during therapy [follow-up (FU)], at a median of 42 days after the first anti-PD-1 antibody dose (**Figure 1A**). Samples were collected between February 2016 and February 2019 at centers in Tübingen and Dresden. Within 24h of donation, peripheral blood mononuclear cells (PBMCs) were isolated using Ficoll-Hypaque density gradient centrifugation and were immediately cryopreserved until use. Patients’ HLA-types were determined using LUMINEX-based high resolution HLA-typing following validated clinical routines (27). Patient characteristics are summarized in **Table 1**. All patients gave their written informed consent for biobanking and use of biomaterials as well as clinical data for scientific evaluation. The Ethics Committee of Tübingen University Hospital approved the study (490/2014BO1, 616/2018BO2).

**Table 1:** Cohort characteristics.

Factor	Category	n	%
Sex	male	24	66.7
	female	12	33.3
Clinical site	Tübingen	34	94.4
	Dresden	2	5.6
Therapy	anti-PD-1	17	47.2
	anti-PD-1 & -CTLA-4	19	52.8
Age	median	68	–
	≥60	22	61.1
	<60	14	38.9
M-category	M1a	2	5.6
(AJCC v7)	M1b	7	19.4
	M1c	24	66.7
	n.a.	3	8.3
HLA-A zygosity	heterozygous	33	91.7
	homozygous	3	8.3
Prior systemic therapies	immunotherapy	6	16.7
	targeted therapy	5	13.9
	chemotherapy	1	2.8
	none	24	66.7
LDH BL	elevated	11	30.6
	normal	25	69.4
LDH FU	elevated	15	41.7
	normal	20	55.6
	unknown	1	2.7

HLA, human leukocyte antigen; LDH, lactate dehydrogenase.

### Determination of MAE-Specific CD8+ T Cells

A library of HLA-A*0201-restricted peptide-loaded MHC (pMHC) multimers (“dextramers”) representing a selection of 167 MAE (**Figure 1B** and **Supplementary Table 1**) was used to screen cryopreserved PBMC samples for the presence of MAE-specific CD8+ T cells. A dextramer consists of a phycoerythrin-labeled dextran backbone to which multiple pMHC complexes are bound. Each dextramer has two 25-mer DNA barcodes, unique for the respective pMHC complex [for technical details see reference (26)]. The nomenclature used here for the MAE-specific CD8+ T cell populations combines the respective protein name and the first 3 letters of the peptide amino acid sequence (e.g., MAGE-A1 KVL represents the MAGE-A1 peptide with the amino acid sequence KVLEYVIKV).

The samples were treated as follows: PBMCs were thawed in batches and a median number of 5 x 10^6^ cells per sample was incubated with the dextramer library. Dead cells were labeled and monoclonal antibodies against CD8 and lineage markers (CD4, CD14, CD16, CD19, CD40) were used to identify CD8+ T cells (**Supplementary Table 2**), followed by overnight fixation using 1% PFA in PBS. The next day, multimer-binding CD8+ T cells were isolated by fluorescence-activated cell sorting (FACS; Melody or Aria fusion, both BD; for gating strategy see **Supplementary Figure 1**). Next, the samples were centrifuged at 5000xg (to break up the cells), supernatant was discarded and the samples were stored as pellets in PBS at -20°C until amplification of the DNA barcodes *via* PCR was carried out. Additionally, an “input” sample (total dextramer library as triplicate) per batch was used to calculate barcode enrichment in individual samples as well as an internal quality control for the amplification of the barcode sequences in each individual sample *via* PCR. The primers employed contained unique DNA sequences (in-house generated “DNA keys”) per patient sample to label the resulting DNA libraries for multiplexed sequencing of pooled samples. The PCR-products were purified (QIAquick PCR purification kit, Qiagen) following the manufacturer´s instructions. The purified samples were then sequenced using the Ion Torrent approach (Thermo Fisher).

Sequencing data were processed by the software package Barracoda, available online at (https://services.healthtech.dtu.dk/service.php?Barracoda-1.8). The tool identifies the DNA barcodes annotated for a given experiment, assigns a sample ID and pMHC specificity to each DNA barcode, and counts the total number of reads and clonally reduced reads for each peptide-MHC-associated DNA barcode. Log_2_FC in read counts mapped to a given sample relative to the mean read counts mapped to triplicate baseline samples are estimated using normalization factors determined by the trimmed mean of M-values method. A minimum read count fraction of 0.1% for a given DNA barcode of the total DNA barcode number in that given sample was set as threshold to avoid false-positive detection of T cell responses due to low number of reads in the baseline samples. DNA barcodes with p<0.01, estimated using the Benjamini–Hochberg method and log_2_FC>1,5 over the input values for the total pMHC library were considered as T cell responses. Barracoda outputs were further processed and annotated using an R-based script. Frequency of a pMHC-specific CD8+ T cell population was estimated based on the %read count of the associated barcode relative to the total %multimer-positive CD8+ T cell population. Sum of the estimated frequency represents the pooled frequencies of all T cell populations in a given sample.

### Visualization of MAE-Specific CD8+ T Cell Signatures and Their Dynamics

The pre-processed sequencing data (as described above) were used to identify and visualize the presence of individual MAE-specific CD8+ T cell populations per sample (output of the Barracoda package). The abundance of the populations within the cohort and the normalized number of detected MAE-specific CD8+ T cell populations provides an overview of all identified T cell clones. It was calculated as the sum of the absolute numbers of each population divided by the number of patients (n=36) for BL and FU samples separately.

Comparing BL and FU samples of each individual patient illustrates the dynamics within the MAE-specific CD8+ T cell signatures under therapy. These dynamics were visualized by the sum of the absolute numbers of detected MAE-specific CD8+ T cell populations per patient in subgroups where either they appeared (i.e. they were not present at BL, but detected at FU), remained stable (present at both time points) or disappeared (present at BL, but no longer detected at FU). The resulting patient-specific vectors (e.g. patient 15 had 24 appearing and 0 disappearing MAE-specific T cell populations under therapy while 3 populations were present at both time points) were designated “melanoma-associated epitope-specific T cell Score A” (T_MAE_S A). The latter was the basis for the calculation of the “melanoma-associated epitope-specific T cell Score B” (T_MAE_S B), which provides a single variable to visualize total changes in MAE-specific CD8+ T cell profiles per patient. T_MAE_S B was calculated by subtracting the sum of disappearing from the sum of appearing MAE-specific CD8+ T cell populations, resulting in a positive value (dominantly “increased” signature), a negative value (dominantly “decreased” signature) or “0” (balance between appearances and disappearances or the lack of MAE-specific CD8+ T cell populations at either timepoint; “balanced” signature). The heatmap was created by Complexheatmaps (28), Circlize (29) and RColorBrewer using R Studio (v1.2.1335).

### Phenotyping of PBMCs

T cell and myeloid compartments were phenotyped for patients with additional cryopreserved PBMC samples available using flow cytometry (n=24 with a T cell antibody panel and n=22 with a myeloid antibody panel). In brief, samples were thawed, dead cells were stained with ethidium monoazide bromide (EMA, Biotinum) and Fcγ receptors were simultaneously blocked using human immunoglobulins (Gamunex, Grifols). For the T cell antibody panel, two aliquots per sample were stained simultaneously with antibodies against the extracellular markers or with the respective isotype-controls. Next, the cells were fixed and permeabilized (eBioscience FoxP3 Transcription Factor Staining Buffer Set, Thermo Fisher Scientific) and stained for FoxP3 expression (**Supplementary Table 3**). For the myeloid cell panel, the samples were stained for cell surface markers (**Supplementary Table 4**).

Samples from both panels were acquired immediately after staining on an LSR II cytometer (BD). Data analysis was performed with FlowJo (v10.7.1, BD), using established gating strategies (**Supplementary Figures 2**, **3**). In brief, single viable lymphocytes were gated for CD3+ T cells. Tregs (CD4+CD25+CD127lowFoxP3+), CD4+ (all CD4+ non-Tregs) and CD8+ T cells were selected for the analysis of checkpoint receptor (TIM-3, LAG-3 and PD-1) and CD25 expression. PD-1 expression was only quantified in BL-samples, as commercially available diagnostic antibody clones cannot reliably stain all PD-1 molecules in patients treated with therapeutic anti-PD-1 antibodies. Myeloid cells were gated as single, viable, lineage-negative (CD3-CD19-CD56-) cells expressing CD11b and CD33. MDSCs were defined as CD14+HLA-DRlow/-, classical monocytes as CD14+CD16-HLA-DR+, intermediate monocytes were defined as CD14+CD16+HLA-DR+ and non-classical monocytes as CD14dimCD16+HLA-DR+.

### Statistical Analyses

OS was defined as the time from the first administration of ICB until death or the end of follow-up. Progression-free survival (PFS) was defined from the start of ICB to the last follow-up or disease progression using RECIST 1.1 criteria (30). Disease-specific survival probabilities (OS and PFS) were analyzed using the Kaplan-Meier method and the respective arms compared using log-rank testing (Prism v5, GraphPad). To test for unintended confounding factors, correlations between clinical parameters and OS were calculated by the confounding function using the swamp R package (31). Changes of the individual immune cell phenotypes under ICB were investigated using the Wilcoxon matched-pairs signed rank test. Group comparisons of immune cell phenotypes between BL and FU were statistically evaluated using the Mann-Whitney U test (Prism v5, GraphPad). Non-parametric Spearman correlations were computed to test for correlations between continuous variables (Prism v5, GraphPad). P<0.05 was considered statistically significant.

To identify dynamic changes of particular MAE-specific CD8+ T cell populations that correlated with patients’ OS, we trained an elastic net regression model (32) on the changes in numbers of MAE-specific T cell populations under therapy (FU sample – BL sample). We computed the elastic net regularization for the Cox models using the glmnet R package (33). To select the elastic net model hyperparameter α (0≤α≤1), the patient cohort was divided into a training and a test set (80% and 20% of the samples, respectively). Here, α=1 is equivalent to a lasso regression, whereas the model reduces to ridge regression with α=0. The best α in [0.1, 1] was selected when we achieved the highest prediction accuracy on the test set. The regularization parameter λ that penalized the least absolute shrinkage (lasso) was selected from 10-fold cross-validation on the training set. The identified MAE-specific CD8+ T cell populations were further investigated for correlations with patients’ OS using uni- and multivariate Cox regressions. Only those T cell populations that revealed robust statistical correlations with patients’ OS in univariate Cox regressions were considered for the calculation of a multivariate Cox proportional hazard model (34). The Cox model was fitted to the data using the survival R package (35).

## Results

### Patients

In this study, the dynamics of 167 MAE-specific CD8+ T cell populations in the peripheral blood of 36 HLA-A*0201+ stage IV melanoma patients under anti-PD-1 ICB were investigated. Blood was drawn before starting therapy and at a median of 42 days thereafter (**Figure 1A**). Median patient age was 68 years (range: 28-88), 66.7% were male and 33.3% were female (n=24 and 12, respectively); 47.2% (n=17) were treated with anti-PD-1 antibody monotherapy, while the remaining 52.8% received a combination of anti-PD-1 and anti-CTLA-4 antibodies (n=19). The one-year OS was 76.1% and the median PFS was 9 months. Cohort characteristics are summarized in **Table 1**.

### Melanoma-Associated Epitope-Specific CD8+ T Cell Signatures

T cells carrying receptors specific for 117 of the 167 MAE-specific dextramers tested were present in at least one sample. We identified T cells specific for a variety of cancer-testis antigens (CTAs), overexpressed antigens and differentiation-specific antigens (36) in 33 of 36 patients. Seventy-two MAE-specific CD8+ T cell populations were present both at baseline (BL) and follow-up (FU) whereas 31 that had not been present at BL appeared at FU; there were also 14 present at BL that were no longer detectable at FU. A qualitative assessment of these 117 shared MAE-specific CD8+ T cell populations revealed a high degree of inter-individual variability suggesting a relatively “private” composition of these T cell signatures, while the observed intra-individual variability reflects ICB-induced effects (**Figure 1B**).

The most prevalent MAE-specific CD8+ T cell population carried receptors for the MAGE-A2 LVH peptide (36.1% of patients at BL and FU), followed by CDKN1A GLG (19.4% at BL and 27.8% at FU) and MAGE-C2 KVL peptide (22.2% at BL and 16.7% at FU). **Figure 1C** depicts the prevalence and the normalized absolute numbers of the 117 detected MAE-specific CD8+ T cell populations at BL and FU. The patterns of MAE-specific CD8+ T cell populations are highly heterogeneous and suggest a patient-unique T cell profile. Similar patterns were also identified for the impact of PD-1 ICB on the estimated frequencies of the individual MAE-specific CD8+ T cell populations (**Supplementary Figure 4** and **Supplementary Table 5**).

### Dynamics of the MAE-Specific CD8+ T Cell Signature Under ICB Correlate With Clinical Outcome

**Figure 2:**
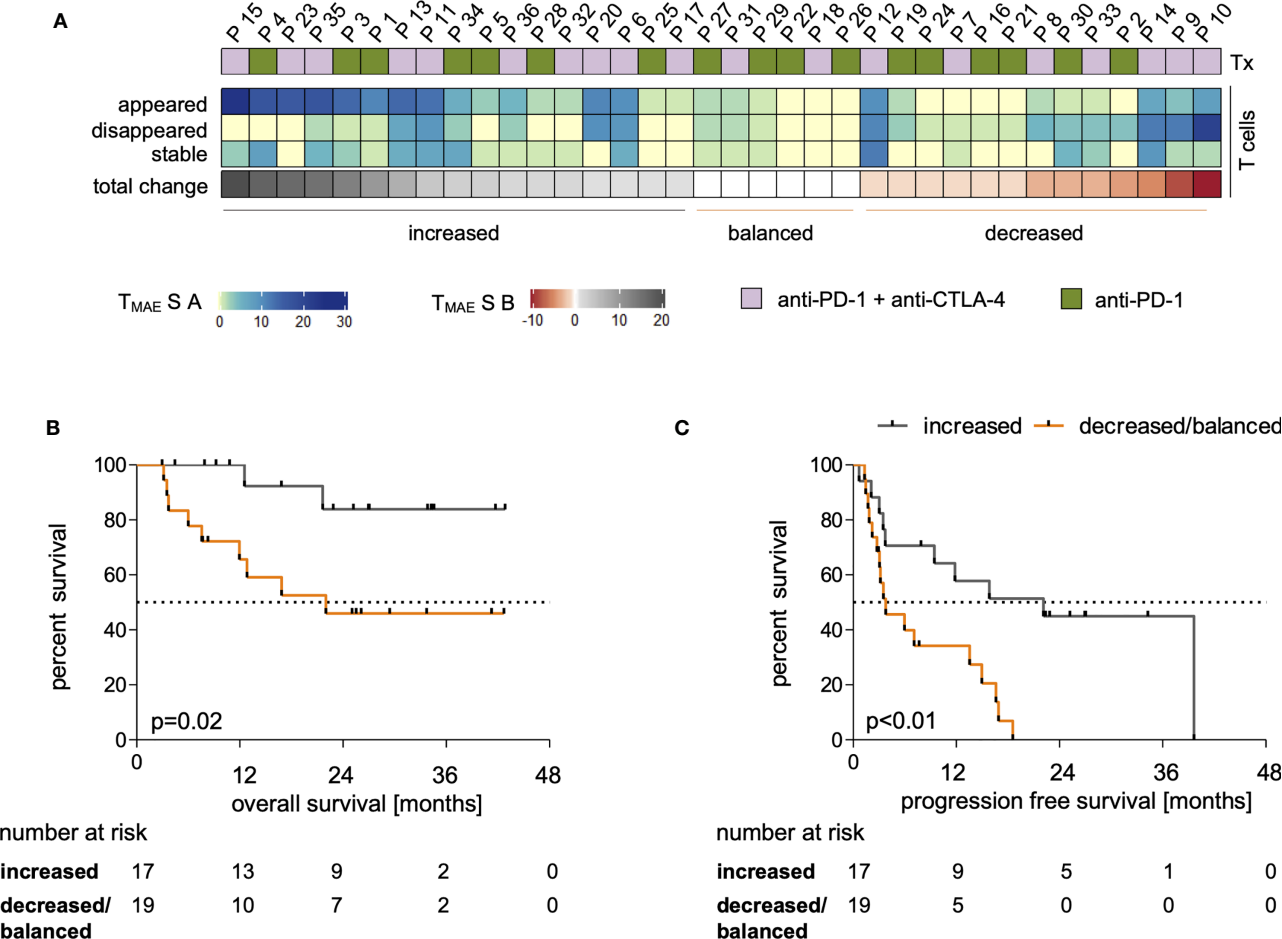
Dynamics within the MAE-specific CD8+ T cell signature correlate with clinical outcome. The applied therapy (Tx) is displayed for each patient in (**A**, top row) and changes within the individual MAE-specific CD8+ T cell signatures under PD-1 ICB are indicated in the absolute number of appearing, disappearing and stable MAE-specific T cell populations summarized as “T_MAE_S A” – a 3 digit score - on a per patient basis (**A**, middle 3 rows). Dominant changes as a single digit value of the MAE-specific CD8+ T cell signature of each patient are displayed in (“T_MAE_S B”) (A, lower row). The cohort was dichotomized for Kaplan-Meier analysis after T_MAE_S B >0 (dominant increased) versus ≤0 (balanced or decreased) T cell signature) and correlated with patients’ OS **(B)** and PFS **(C)**.

Dynamics of the investigated MAE-specific CD8+ T cell signatures were investigated in patients receiving PD-1 ICB alone or in combination with CTLA-4 antibodies (**Figure 2A**). To assign ICB-driven dynamics to the individual patient, we defined an MAE-specific CD8+ T cell score A (T_MAE_S A) that summarizes the individual numbers of i) appearing, ii) disappearing or iii) stable MAE-specific CD8+ T cell populations in the blood under ICB (**Figure 2A**). We identified patients with dominantly increasing, dominantly decreasing, or with little or no change in the total MAE-specific CD8+ T cell T_MAE_S A. These three parameters were then used to define the score T_MAE_S B reflecting the dominance of either an increasing or not-increased (decreased or balanced) MAE-specific CD8+ T cell signature (**Figure 2A**).

Dichotomizing the cohort according to T_MAE_S B as defined above allowed a correlation to be made with clinical outcome. We found that a T_MAE_S B >0 (dominantly increased CD8+ MAE-specific T cell signature) was associated with both prolonged OS (p=0.02, HR:0.24, **Figure 2B**) and PFS (p<0.01, HR:0.3, **Figure 2C**). Not only the qualitative, but also a semi-quantitative analysis identified a significant increase of the estimated frequencies of the CD8+ MAE-specific T cell populations in patients with a T_MAE_S B >0 (p=0.04), but not in those with a T_MAE_S B ≤0 (p=0.39) (**Supplementary Figure 5**). The swimmer plot in **Supplementary Figure 6** summarizes further clinical follow-up data for each individual patient.

Major demographic and clinical factors (age, sex, type of therapy, elevated LDH and previous systemic therapies), that might confound the identified associations of MAE-specific CD8+ T cell signatures and dynamics under ICB with clinical outcome were correlated with each other. The confounding function matrix revealed no concerning correlations with OS and PFS (**Supplementary Figure 7**). By univariate analyses, there were also no statistically significant differences in OS or PFS between patients receiving anti-PD-1 antibodies alone or in combination with anti-CTLA-4 antibodies (**Supplementary Figure 8A**); there were also no correlations with age (**Supplementary Figure 8B**). Furthermore, there was no significant correlation between age and T_MAE_S B (**Supplementary Figure 8C**). Thus, the identified dominantly increasing MAE-specific CD8+ T cell signatures under ICB (T_MAE_S B >0) in patients with prolonged OS and PFS can be considered as not confounded by typical clinical and demographic variables.

### Alterations in Cellular Phenotypes Correlate With the Dynamics of MAE-Specific CD8+ T Cell Signatures Under ICB

**Figure 3:**
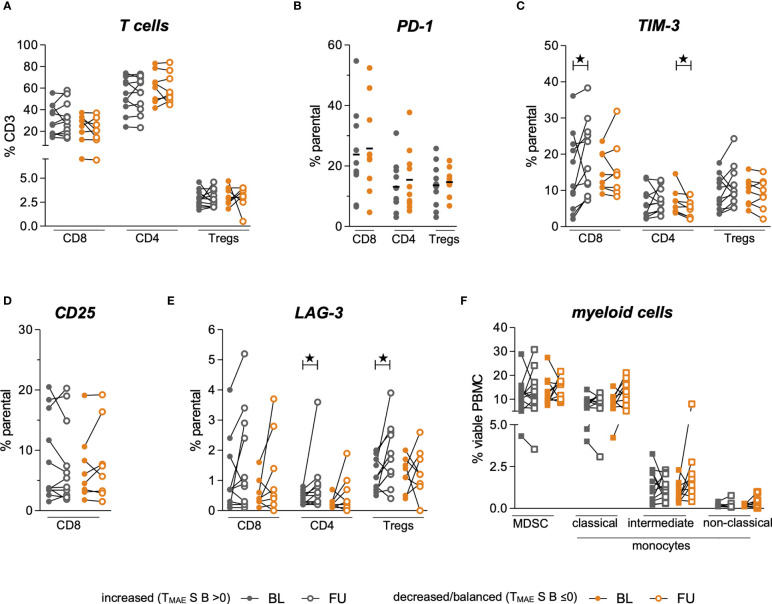
Comparison of myeloid and T cell phenotypes in a group of patients dichotomized by T_MAE_S (B) Baseline (BL, full symbol) and follow-up samples (FU, empty symbols) of each individual patient are connected by a line. Frequencies of patients with an increased MAE-specific CD8+ T cell T_MAE_S B are displayed in grey, those with a decreased/balanced in orange. Frequencies of Tregs, CD8+ and CD4+ T cells among all CD3+ T cells **(A)**. Frequency of PD-1-expressing subsets at baseline **(B)**. Alterations of frequencies of TIM-3-expressing **(C)**, CD25-expressing **(D)** and LAG-3-expressing **(E)** T cell subsets. Changes of frequencies of MDSC and monocytic phenotypes are shown in **(F)** ★ indicate p-values <0.05.

To study the associations of the expression profiles of checkpoint molecules on T cells and the abundance of immune regulatory cells in the context of the above-described beneficial increase of the individual MAE-specific CD8+ T cell signatures (T_MAE_S B >0), we assessed phenotypic profiles of myeloid cells and T cells. These phenotypes were comparatively evaluated in patients with increased (T_MAE_S B >0) or decreased/balanced (T_MAE_S B ≤0) MAE-specific CD8+ T cell signatures, as defined above. We found no differences between the two patient groups at BL for any of the observed immune cell phenotypes (**Figures 3A–F**), including frequencies of PD-1+ cells within CD8+ T cells, CD4+ T cells and Tregs (**Figure 3B**). There were also no differences regarding changes of total CD8+ T cells, CD4+ T cells or Treg frequencies (**Figure 3A**). However, patients with an increasing MAE-specific CD8+T cell signature under ICB had increasing frequencies of TIM-3+CD8+ T cells (p=0.04, **Figure 3C**), whereas no differences were found in the CD25+CD8+ T cell population (**Figure 3D**). Furthermore, we observed that a LAG-3+ subset of the CD4+ and Treg populations increased significantly in patients with an increasing MAE-specific CD8+ T cell signature (p=0.04 and p=0.01, respectively, **Figure 3E**) but not in the reciprocal group. Vice versa, patients with a decreased/balanced MAE-specific CD8+ T cell signature exhibited a decrease in the frequency of TIM-3+CD4+ T cells (p=0.02, **Figure 3C**).

The frequencies of MDSCs, intermediate, classical- and non-classical monocytes revealed no statistically significant changes under ICB either in patients with increasing or with decreasing/balanced MAE-specific CD8+ T cell signatures (**Figure 3F**). The medians and interquartile ranges (IQR) of all these populations are shown in **Table 2**.

**Table 2:** Median and IQRs of the determined immune phenotypes, dichotomized by T_MAE_S B (>0: increased vs ≤0: decreased/balanced).

Cell subset	Time point	Increased		Decreased/balanced	
		Median	IQR	Median	IQR
CD8+	BL	26.6	26.1	27.3	18.0
	FU	29.2	27.5	20.7	18.1
CD4+	BL	55.3	27.6	62.4	27.0
	FU	56.1	28.7	55.3	25.5
Tregs	BL	2.9	1.3	3.0	1.5
	FU	2.6	1.7	3.2	1.3
PD1+CD8+	BL	19.7	19.5	23.0	23.0
PD1+CD4+	BL	11.0.	11.6	11.9	14.4
PD1+Tregs	BL	13.6	13.1	14.4	4.6
TIM-3+CD8+	BL	11.2	17.9	13.3	9.3
	FU	16.0	17.8	14.2	10.1
TIM-3+CD4+	BL	5.8	5.6	6.2	4.5
	FU	6.6	5.8	5.2	4.0
TIM-3+Tregs	BL	7.7	8.3	10.3	6.1
	FU	9.8	7.8	9.3	6.6
CD25+ CD8+	BL	3.8	13.6	5.3	6.8
	FU	5.4	12.3	6.6	11.2
LAG-3+CD8+	BL	0.7	1.6	0.4	0.6
	FU	0.9	2.7	0.6	2.2
LAG-3+CD4+	BL	0.4	0.4	0.2	0.2
	FU	0.6	0.6	0.3	0.8
LAG-3+Tregs	BL	0.7	1.6	0.4	0.6
	FU	0.9	2.7	0.6	2.2
MDSC	BL	10.8	6.7	11.6	8.1
	FU	11.3	9.5	16.5	7.3
Classical monocytes	BL	7.8	3.0	9.2	4.3
	FU	9.0	4.9	12.1	10.0
Intermediate monocytes	BL	1.1	1.7	0.8	0.9
	FU	1.1	1.4	1.6	1.3
Non-classical monocytes	BL	0.2	0.2	0.2	0.2
	FU	0.2	0.3	0.4	0.4

BL, baseline; FU, follow-up; IQR, interquartile range; MDSC, myeloid derived suppressor cells.

### Regression-Based Identification of MAE-Specific CD8+ T Cell Populations Correlating With OS

**Figure 4:**
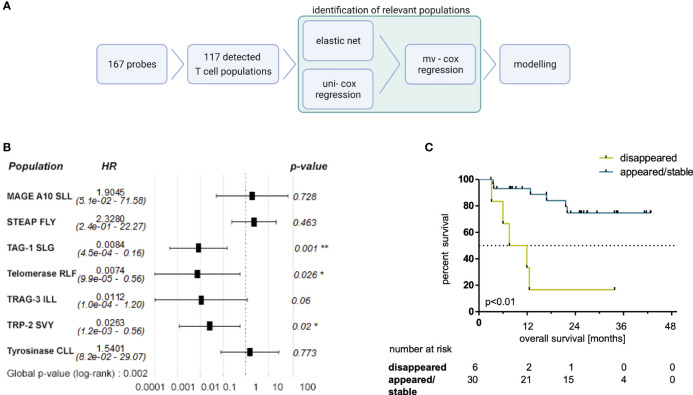
Selected MAE-specific CD8+ T cell populations identified by a regression approach correlate with clinical outcome. The applied workflow to identify relevant MAE-specific T cell populations **(A)** (created with BioRender.com) and the resulting hazard ratios (HR) (including the 95% confidence intervals) of the identified MAE-specific T cell populations are shown in a forest plot **(B)**. Those CD8+ T cell populations, specific for TAG-1 SLG, Telomerase RLF and TRP2 SVY, independently correlating with OS, were combined in a comprehensive model. Patients with a disappearance of at least one of those populations had a shorter OS **(C)** compared to the reciprocal group. * indicate p-values <0.05 and ** indicate p-values <0.01.

Next, we aimed to identify the most relevant dynamics of certain MAE-specific CD8+ T cell populations through correlations with patients’ OS. We first noticed that similar to the previously studied NY-ESO-1 TAA (25), the disappearance of NY-ESO-1 QLS and SLL-specific T cells from the periphery tended to correlate with a prolonged OS under ICB (p=0.14; data not shown). Next, we performed several regression analyses in parallel and/or in sequence (**Figure 4A**) to identify those MAE-specific T cell populations with the greatest relevance for therapy outcome in an unbiased approach. First, an elastic net model was trained to identify associations of the dynamics of MAE-specific CD8+ T cell populations with patients’ OS. The resulting model achieved the highest prediction accuracy as well as a smaller set of features at an α of 0.7 (**Supplementary Table 6**). Dynamics of 9 of the 117 detected MAE-specific CD8+ T cell populations (the CTAs MAGE-A10 SLL, TAG-1 SLG, TRAG-3 ILL, the differentiation antigen TRP-2 SVY and the overexpressed antigens STEAP1 FLY, P-cadherin FII, Telomerase RLF, Telomerase ILA and Tyrosinase CLL) were identified as most informative for predicting clinical outcome by regression analysis. Univariate Cox regression analysis using Wald and log-rank testing identified the dynamics of two of these 9 MAE-specific CD8+ T cell populations as potentially unreliable and they were excluded from further modelling (P-cadherin FII and Telomerase ILA; see **Supplementary Table 7**). Multivariate Cox regression analysis of the remaining 7 MAE-specific CD8+ T cell populations revealed independent correlations of the dynamics of CD8+ T cells specific for TAG-1 SLG, Telomerase RFL and TRP-2 SVY (p<0.01, HR:0.01, p=0.03, HR:0.01; and p=0.02, HR:0.03, respectively; **Figure 4B**). A combinatorial model, comprising the dynamics of these 3 MAE-specific CD8+ T cell populations suggested that patients exhibiting a disappearance from the peripheral blood of at least one of these MAE-specific CD8+ T cell populations under therapy had a significantly shorter OS compared to those patients with appearing or stable T cell populations (p<0.01, HR:40.96, **Figure 4C**). Importantly, the disappearance of these selected MAE-specific CD8+ T cells, did not correlate with any observed dynamics of all other investigated MAE-specific CD8+ T cell populations (T_MAE_S A or B) at the individual patient level (P35, P34, P29, P12, P16, P9).

Furthermore, patients in the appearing/stable group also experienced an increase in the sum of estimated frequencies of these three T cell populations specific for TAG-1 SLG, Telomerase RFL and TRP-2 SVY (**Supplementary Figure 9A**; p=0.03). Interestingly, the sum of the estimated frequencies of these three MAE-specific CD8+ T cell populations was higher at BL in patients with a disappearance of at least one of them under therapy compared to the reciprocal group (**Supplementary Figure 9A**; p<0.01). No significant differences were found in this subset-analysis applying the sum of estimated frequencies of all detected MAE-specific CD8+ T cell populations (**Supplementary Figure 9B**).

Taken together, these results underscore the importance of the composition, the frequency and the dynamics of the individual peripheral anti-melanoma T cell repertoire early under ICB.

## Discussion

Here, we applied a high-throughput approach to investigate the *ex vivo* dynamics of peripheral blood melanoma-associated epitope-specific CD8+ T cell signatures in HLA-A*0201+ stage IV melanoma patients under ICB. To this end, using multiple pMHC dextramers, we investigated 167 MAE-specific CD8+ T cell populations of which 117 were found to be present in one or more patients at one or more time points. We observed T cell recognition most prevalent towards an epitope (LVH) derived from the differentiation antigen MAGE-A2, which was detected in over one-third of the cohort. Also, other previously-identified MAE-specific CD8+ T cell populations such as Melan-A ELA were detected in about 20% of pre-treatment baseline as well as follow-up samples. Other tumor-associated epitopes such as Telomerase RLF or p53 RMP were less frequently recognized. In agreement with expectations, the distribution of the abundance of the different MAE-specific CD8+ T cell populations varied greatly between BL and FU, and the identified individual signatures were heterogeneous and often private (37). On a per-patient level, the dynamics of MAE-specific CD8+ T cell populations were of particular interest. On the basis of these dynamics, we defined two scores. One of these, T_MAE_S A consisted of 3 variables reflecting the number of appearing, disappearing and stable MAE-specific CD8+ T cell populations. Secondly, based on T_MAE_S A we defined T_MAE_S B as a single variable reflecting a dominant increasing or not-increasing (decreasing or balanced) T cell signature. As expected, we found ICB-associated dynamics of MAE-specific CD8+ T cell signatures in the majority of the observed patients, regardless of whether they received PD-1 monotherapy or a combination of PD-1 and CTLA-4 antibodies, similar to reports on the dynamics of selected TAA- or virus-specific T cells under PD-1 ICB (11, 12, 38) or neo-epitope specific T cells under PD-L1 ICB (39). However, our findings contrast with those of a recent study from Gangaev et al. who reported a broadening of MAE-specific CD8+ T cells mostly in patients treated with anti-CTLA-4 antibodies (but only rarely under PD-1 monotherapy) (37). Despite highly overlapping pMHC-multimer panels in both studies, the discrepancy may be explained by the different multimer approaches employed. Thus, we detected 117 MAE-specific CD8+ T cell populations using 167 dextramers while Gangaev et al. found 7 MAE-specific CD8+ T cell populations in anti-PD-1 and 6 in anti-CTLA-4 treated patients using 71 tetramers. Admittedly, both studies enrolled a modest number of patients (36 in the current study-vs-9 in the anti-CTLA-4- and 24 in the anti-PD-1-treated cohorts in the work of Gangaev et al.). Thus, future investigations of MAE-specific CD8+ T cells under ICB are warranted.

The main finding of the present study was that dominantly increasing MAE-specific CD8+ T cell signatures summarized in T_MAE_S B correlated with prolonged OS and PFS. This complements published data that associates epitope spreading (40) or induction of TCR repertoire divergence (41) under ICB with beneficial clinical outcomes. ICB-induced epitope spreading and disease control requires the patient´s possession of an appropriate T cell receptor repertoire and retention of T cell functionality. Additionally, the presence of regulatory immune cells such as MDSCs, or Tregs that might dampen anti-cancer T cell responses can play a critical role in cancer immunotherapy (42, 43). To evaluate the impact of the abundance of such immune regulatory cells in the context of the above-described beneficial increase of the individual MAE-specific CD8+ T cell score B (T_MAE_S B), we assessed phenotypic MDSC and Treg data, and also checkpoint receptor expression on T cell subsets in our cohort. Our findings revealed no differences between patients with dominantly decreasing/balanced (T_MAE_S B ≤0) or increasing (T_MAE_S B >0) MAE-specific CD8+ T cell signatures before the start of ICB (BL) in any of the observed cellular phenotypes including frequencies of PD-1+, LAG-3+ and TIM-3+ cells within Tregs, CD8+ and CD4+ T cell populations. Perhaps this is not surprising, despite reports that PD-1+ peripheral blood T cell subsets harbor tumor-specific T cell populations (44), because the expression of PD-1, LAG-3 and TIM-3 alone has not been identified as a predictive biomarker candidate for successful ICB. Nonetheless, recent reports suggest that combinatorial analyses of several such checkpoint receptor-expressing T cell subsets may reveal an association with clinical outcome. For example, the PD-1+CD8+/PD-1+Treg ratio in tumor-resident cells was reported to predict the clinical efficacy of PD-1 ICB (45), and a combinatorial analysis of LAG-3 expression on several peripheral blood cell subsets (prominently on CD8+ T cells) before the start of PD-1 ICB correlated with poorer clinical outcome (46). Also the utility of examining relationships between phenotypes and clinical features under ICB, such as a validated negative association with OS of the expression of Ki67 on circulating PD-1+ CD8+ T cells and tumor burden has been reported (12).

However, comparative analyses of alterations in the observed T cell phenotypes under PD-1 ICB identified significant differences in our cohort that were dichotomized according to their T_MAE_S B. We observed an increase of TIM-3+CD8+ T cells in patients with (T_MAE_S B >0) but not in those without (T_MAE_S B ≤0) an increasing MAE-specific CD8+ T cell signature, which might reflect a PD-1 blockade-driven evasion of co-inhibitory signaling through the TCR complex for negative regulatory checkpoint receptors other than TIM-3. The latter mechanism has been described in a mouse model of lung adenocarcinoma, where adaptive resistance to anti-PD-1 treatment was associated with an upregulation of TIM-3 on PD-1+ T cells in the tumor (47). Whether such an increase in the TIM-3+CD8+ T cell population in our study correlates with exhaustion of CD8+ T cells (48) or rather marks competent/reactive CD8+ T cells could not be functionally assessed here. The dynamics of changes of CD8+ T cells expressing the activation marker CD25 did not allow further conclusions either. Increases in LAG-3+CD4+ T cells and LAG-3+ Tregs in patients with increasing MAE-specific CD8+ T cell signatures, but also a decrease of TIM-3+CD4+ T cells in patients with decreasing/balanced MAE-specific CD8+ T cell signatures, might additionally illustrate the multifaceted modulatory effects of ICB on the CD4+ as well as the CD8+ T cell population, which could not be examined in detail here. In particular, the increases of LAG-3+CD4+ T cell and LAG-3+Treg frequencies in patients with an increasing MAE-specific CD8+ T cell signature underscores our hypothesis that these patients have retained a competent T cell compartment and therefore have a better chance of obtaining clinical benefit through ICB. This hypothesis is consistent with data from a study by Zelba et al. who reported an increase of LAG-3+ and TIM-3+ CD4+ and CD8+ T cells in an *in-vitro* PD-1 ICB assay in renal cell carcinoma TILs (49). Unexpectedly, we did not see differences in monocytic and MDSC subsets between the two patient groups, suggesting no direct associations between dynamics of MAE-specific CD8+ T cell populations and the peripheral frequencies of these cells that were previously reported as biomarker candidates in melanoma under ICB (8, 9).

We exploited our dataset further by applying a regression-based approach to identify those MAE-specific CD8+ T cell populations that were most informative for OS – the most robust endpoint of our study. We found that a loss of CD8+ T cells specific for the differentiation antigen TRP-2 SVY, the CTA TAG-1 SLG and the overexpressed antigen Telomerase RLF was associated significantly and independently with shorter OS. The resulting combinatorial model defined a subgroup of patients with a significantly reduced OS, characterized by a loss of at least one of the three MAE-specific CD8+ T cell populations. Reasons for the disappearance of TAA-specific T cells – and thus also the loss of these mostly apparently clinically-beneficial MAE-specific CD8+ T cells - from the periphery might be diverse. This might be of particular relevance as we recently reported the early disappearance of functional Melan-A- or NY-ESO-1-reactive CD4+ and/or CD8+ T cells from the peripheral blood in some melanoma patients with superior OS and PFS under PD-1 ICB resulting from a hypothetical migration to the metastases (25). A similar pattern for the dynamics of NY-ESO-1-specific CD8+ T cells was also found in the present study. However, the (opposite) correlation of the loss of T cells specific for the TRP-2 SVY-, TAG-1 SLG- and Telomerase RLF-peptide-MHC complexes with OS noted here might be explained by these cells being dysfunctional or exhausted already before the start of ICB, as commonly reported in advanced stages of cancer (50). Taken together, we hypothesize that T cell specificity (37), kinetics of (ICB triggered) epitope accessibility (40) and essentially also functionality (20–22, 24, 25) at the single cell level might be considered as major features to classify T cell populations that actively contribute to cancer immunosurveillance as opposed to those from dysfunctional and/or anergic subsets that cannot be reinvigorated by ICB. Thus, such future evaluations of TAG-1-, Telomerase- and TRP-2-reactive T cells (including also CD4+ T cells) is warranted to discriminate between functionally competent and dysfunctional specific T cell clones, similar to our previous studies on MAGE-A3, Survivin-, Melan-A- and NY-ESO-1-reactive T cell populations (7, 20, 21).

Taken together, our pilot study shows a high degree of individuality in the MAE-specific CD8+ T cell profiles in these melanoma patients. Nevertheless, we were able to identify some epitopes that might contribute to the search for targets for novel TAA-based cancer vaccines, which are currently regaining attention (19, 51, 52). However, this requires further in-depth studies of these epitopes and in addition to the qualitative investigations described in this pilot study, must also include functional studies. Furthermore, our results provide important insights into the dynamics of circulating MAE-specific CD8+ T cells under ICB and should contribute to a better understanding of the role of these cells in cancer rejection.

## Data Availability Statement

The raw data supporting the conclusions of this article will be made available by the authors, without undue reservation.

## Ethics Statement

The studies involving human participants were reviewed and approved by Ethics Committee of Tübingen University Hospital. The patients/participants provided their written informed consent to participate in this study.

## Author Contributions

KW-H, BW, and SH contributed to conceptualization. AG, SB, SH, and KW-H contributed to the methodology. AG, SH, and KW-H were administering this project. AG, TM, CH, ST, JB, JS, SH, and KW-H were involved in investigation. AG, TM, CH, ST, SH, and KW-H contributed to data curation, formal analysis and investigation. AG, SB, TM, SH, and KW-H were involved in formal analysis and visualization. TA, NW, RK, FM, CG, and TE were providing resources and funding was acquired by CG, BW, KW-H, and SH. This project was supervised by TE, GP, MC, KW-H, and SH. AG, GP, and KW-H wrote the first draft of the manuscript. SB and SH wrote sections of the manuscript. All authors contributed to manuscript revision, read and approved the submitted version.

## Funding

This work was partially funded by the Medical Faculty of the University of Tübingen (2509-0-0), Bristol-Myers Squibb (CA209-9P4) and the Klaus Tschira Foundation (00.316.2017) (to KW-H). Additionally, it was in part supported by the European Research Council, StG 677268 NextDART, the Lundbeck Foundation Fellowship R190–2014–4178 and the Carlsberg foundation (to SH).

## Conflict of Interest

CG reports receiving commercial research grants from Bristol-Myers Squibb, Novartis, and Roche, and is a consultant/advisory board member for Amgen, Bristol-Myers Squibb, Merck Sharp & Dohme, Novartis, and Roche. BW reports receiving commercial research grants from, is a consultant/advisory board member for, and reports receiving travel reimbursement from Bristol-Myers Squibb and Merck Sharp & Dohme. FM reports receiving commercial research grants from Novartis and Roche, and has received travel support or/and speaker´s fees or/and advisor´s honoraria by Novartis, Roche, Bristol-Myers Squibb, Merck Sharp & Dohme, and Pierre Fabre. NW reports an advisory role for Pierre-Fabre and Sanofi, consultant’s honoraria from Novartis, and has received travel support from AbbVie and Amgen outside the submitted work. GP has received speaker´s honoraria from Novartis, Roche, Pfizer, GlaxoSmithKline and Astellas. KW-H received commercial research grants from the CatalYm GmbH and travel support from SITC (Society for Immunotherapy of Cancer). SH is the cofounder of Immumap, Tetramer-shop and PokeAcell and is the co-inventor of the patents WO2015185067 and WO2015188839 for the barcoded MHC technology which is licensed to Immudex. The data presented in this study is not directly involved in these activities. TE has received travel support or/and speaker´s fees or/and advisor´s honoraria by Sanofi, Novartis, Bristol-Myers Squibb, Merck Sharp & Dohme, Almiral Hermal, and Pierre Fabre. TA reports institutional grants from SkylineDx, institutional grants and personal fees from Novartis, institutional grants from NeraCare, personal fees from BMS, institutional grants from Sanofi, personal fees from CeCaVa, personal fees from Pierre Fabre, outside the submitted work.

The remaining authors declare that the research was conducted in the absence of any commercial or financial relationships that could be construed as a potential conflict of interest.

## Publisher’s Note

All claims expressed in this article are solely those of the authors and do not necessarily represent those of their affiliated organizations, or those of the publisher, the editors and the reviewers. Any product that may be evaluated in this article, or claim that may be made by its manufacturer, is not guaranteed or endorsed by the publisher.
