# Supplementary material for: Dynamics of Melanoma-Associated Epitope-Specific CD8+ T Cells in the Blood Correlate With Clinical Outcome Under PD-1 Blockade
Source: Front Immunol. 2022 Jul 7;13:906352. doi: 10.3389/fimmu.2022.906352 (PMC9300827; doi:10.3389/fimmu.2022.906352)
Supplement: Supplementary file 1 [file DataSheet_1.pdf]

Dynamics of melanoma-associated epitope-specific CD8+ T cells in the blood correlate with clinical outcome under PD-1 blockade

Andrea Gaißler, Trine Sundebo Meldgaard, Christina Heeke, Sepideh Babaei, Siri Amanda Tvingsholm, Jonas Bochem, Janine Spreuer, Teresa Amaral, Nikolaus Benjamin Wagner, Reinhild Klein, Friedegund Meier, Claus Garbe, Thomas Eigentler, Graham Pawelec, Manfred Claassen, Benjamin Weide, Sine Reker Hadrup*, Kilian Wistuba-Hamprecht*

* shared last authorship

supplementary material

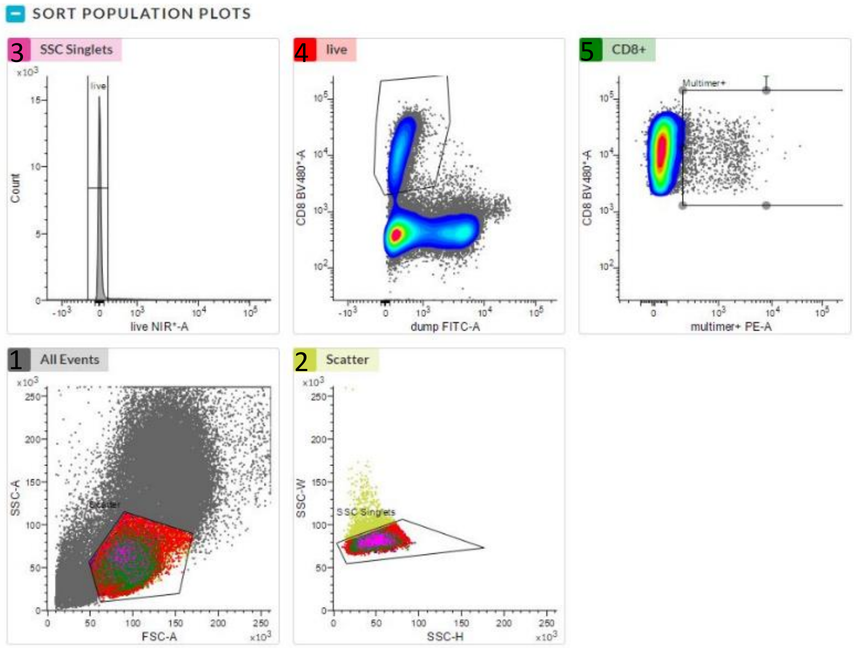

Supplementary Figure 1: Gating strategy for fluorescence activated cell sorting of MAE-specific CD8+ T cells.

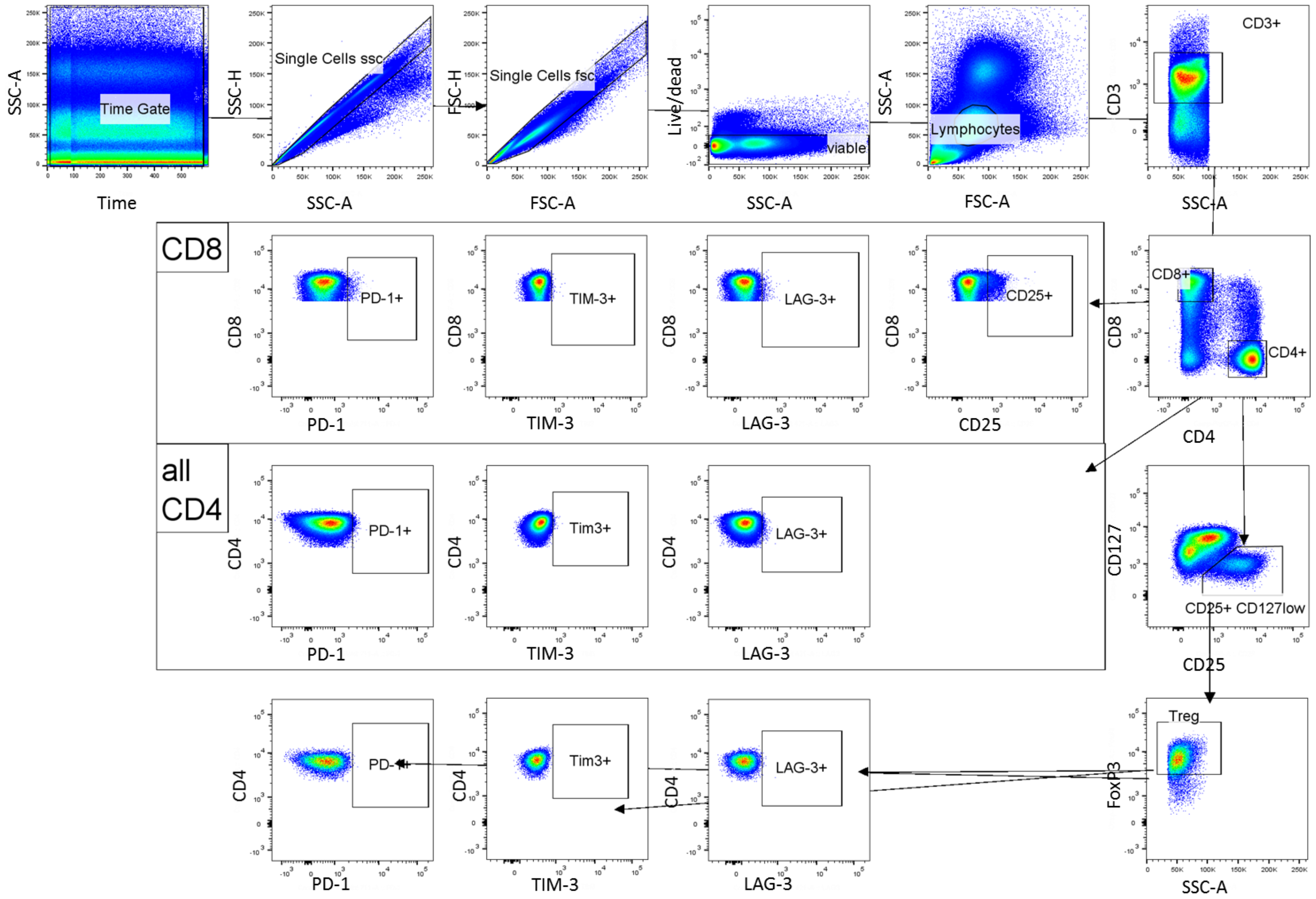

Supplementary Figure 2: Gating strategy for phenotypic assessment of peripheral blood T cells. Gating for checkpoint molecules (PD-1, LAG-3 and TIM-3) was established on the basis of isotype controls on each sample**.**

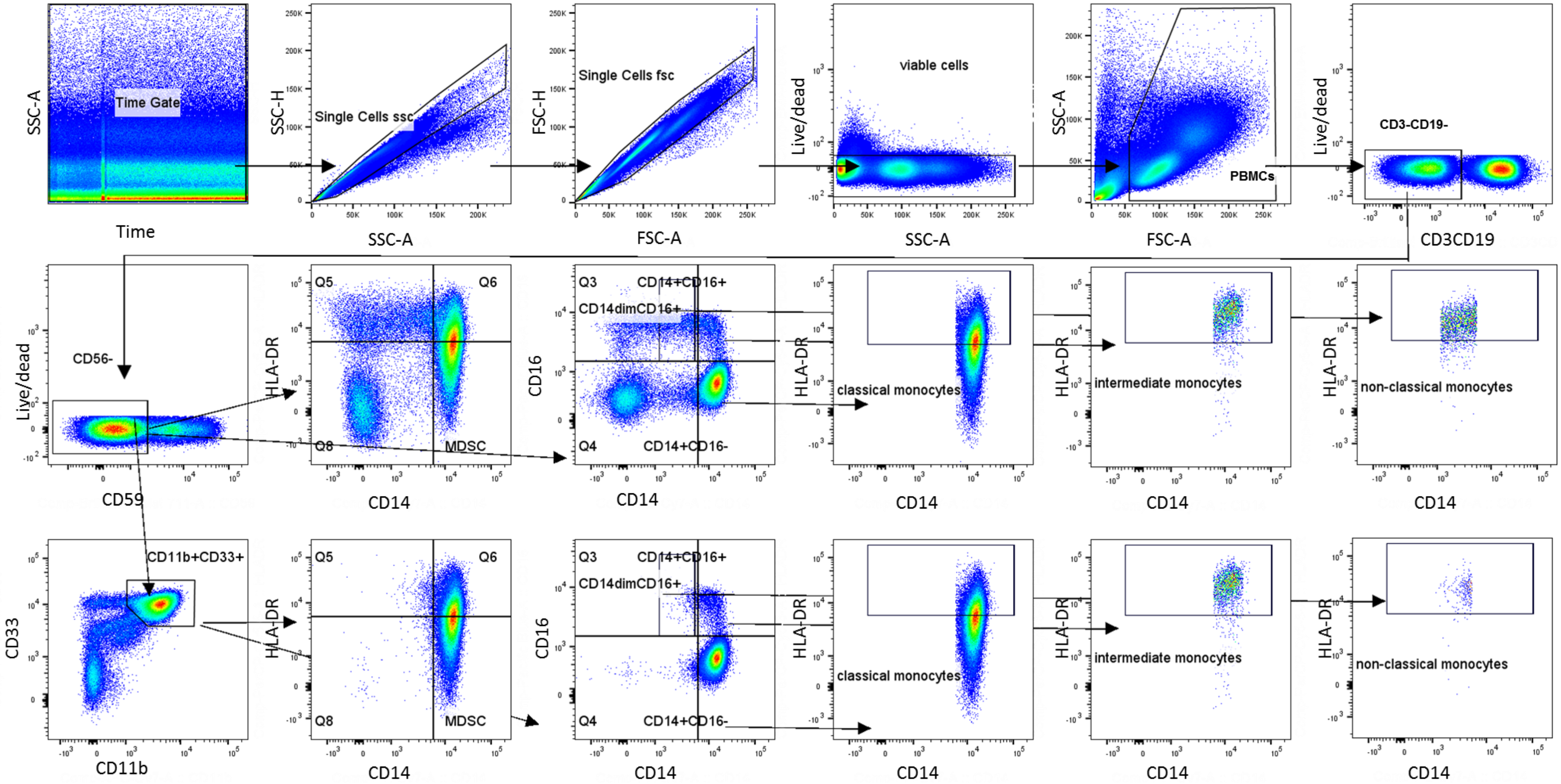

Supplementary Figure 3: Gating strategy for phenotypic assessment of myeloid cells. Gates of MDSC, classical- intermediate- and non-classical monocytes were set on CD3-CD19-CD56- cells (middle panel) and then copied on the CD11b+CD33+ population (lower panel).

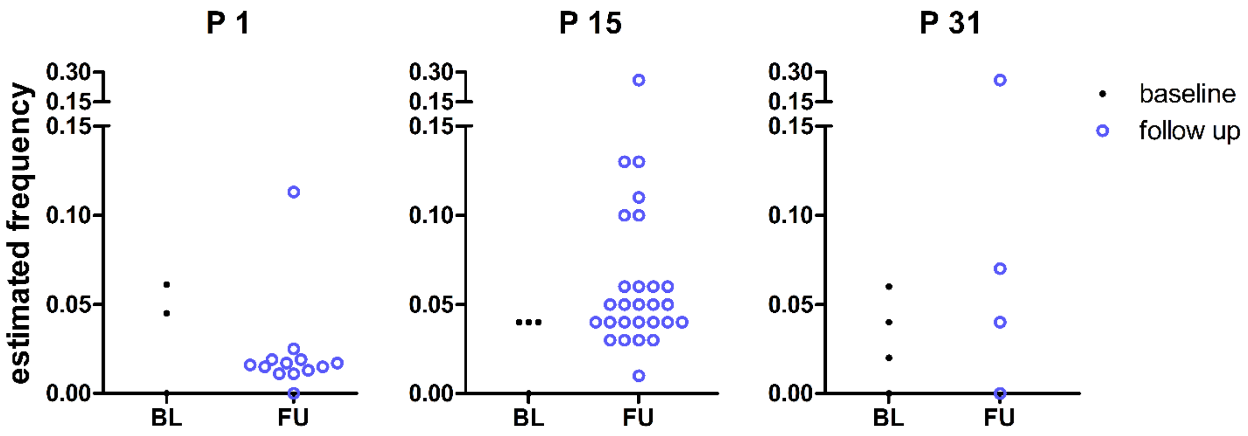

Supplementary Figure 4: Estimated frequency of MAE-specific CD8+ T cell populations in representative patients at BL and FU.

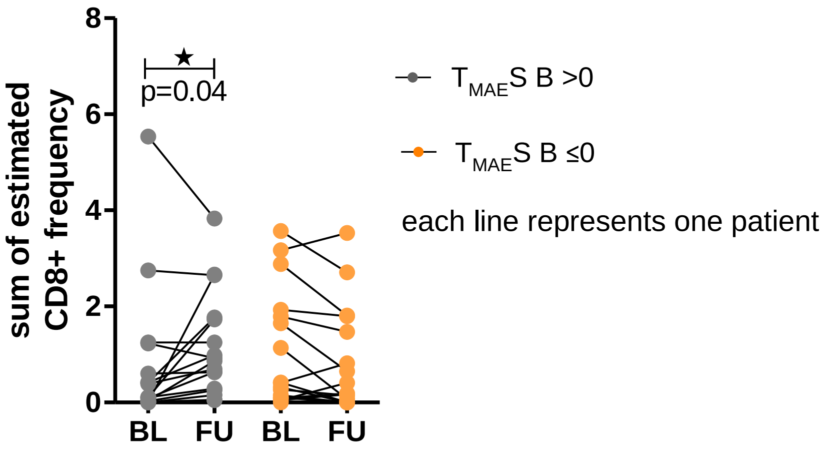

Supplementary Figure 5: Sum of estimated frequencies of all MAE-specific CD8+ T cell populations relative to all CD8+ T cells. Each line, that connects two dots represents one patient. Patients with an $T_{MAE}S$ B >0 (dominantly increasing signature) are displayed with grey symbols, while those with an $T_{MAE}S$ B ≤0 (balanced or dominantly decreasing signature) are illustrated using orange symbols.

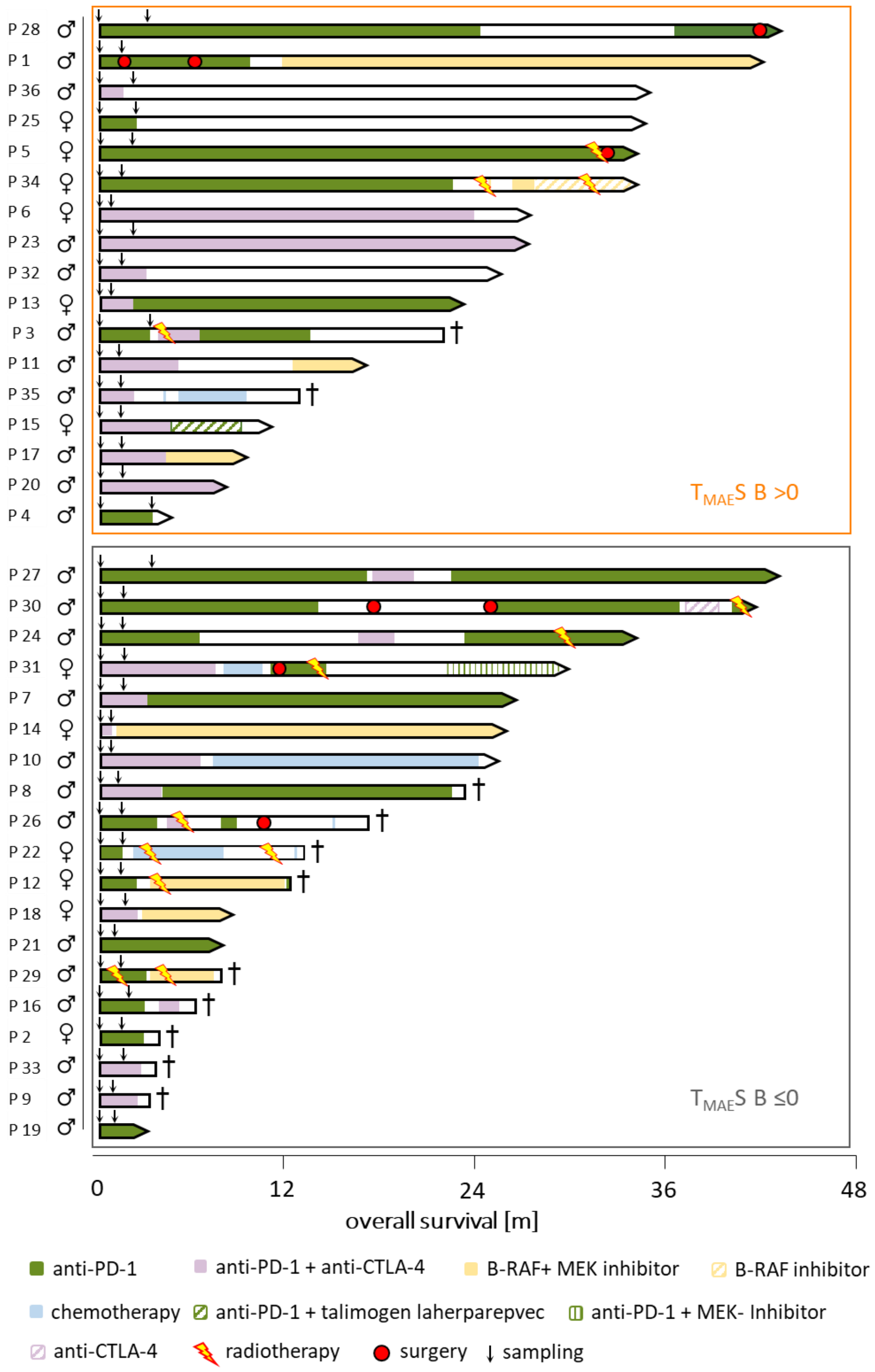

Supplementary Figure 6: Overview of the clinical follow up of the individual patients in this study. The swimmer plot illustrates sampling, therapies and clinical follow up until last contact or death of each individuum. The cohort is dichotomized by dynamics of the melanoma-associated epitope (MAE)-specific CD8+ T cell populations, defined by T_{MAE}S B. This score reflects either a dominantly increasing (T_{MAE}S B >0) or a dominantly decreasing or stable (T_{MAE}S B ≤0) T cell signature under therapy.

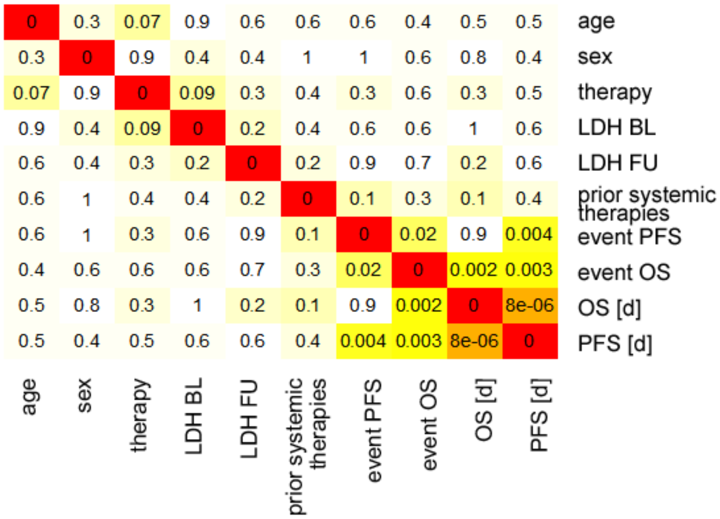

Supplementary Figure 7: ‘Confounding matrix’ displaying p-values of the individual correlations of potentially confounding demographic and clinical data. Heatmap colors represent the p-value of the chi-square test. Data description: LDH: lactate dehydrogenase levels in patients’ serum, divided by diagnostic cut-off; prior systemic therapies: yes/no and included prior immunotherapy, kinase inhibition, and chemotherapy; event PFS/OS: whether the patient had a progress or deceased; OS [d]: overall survival in days; PFS [d]: progression free survival in days

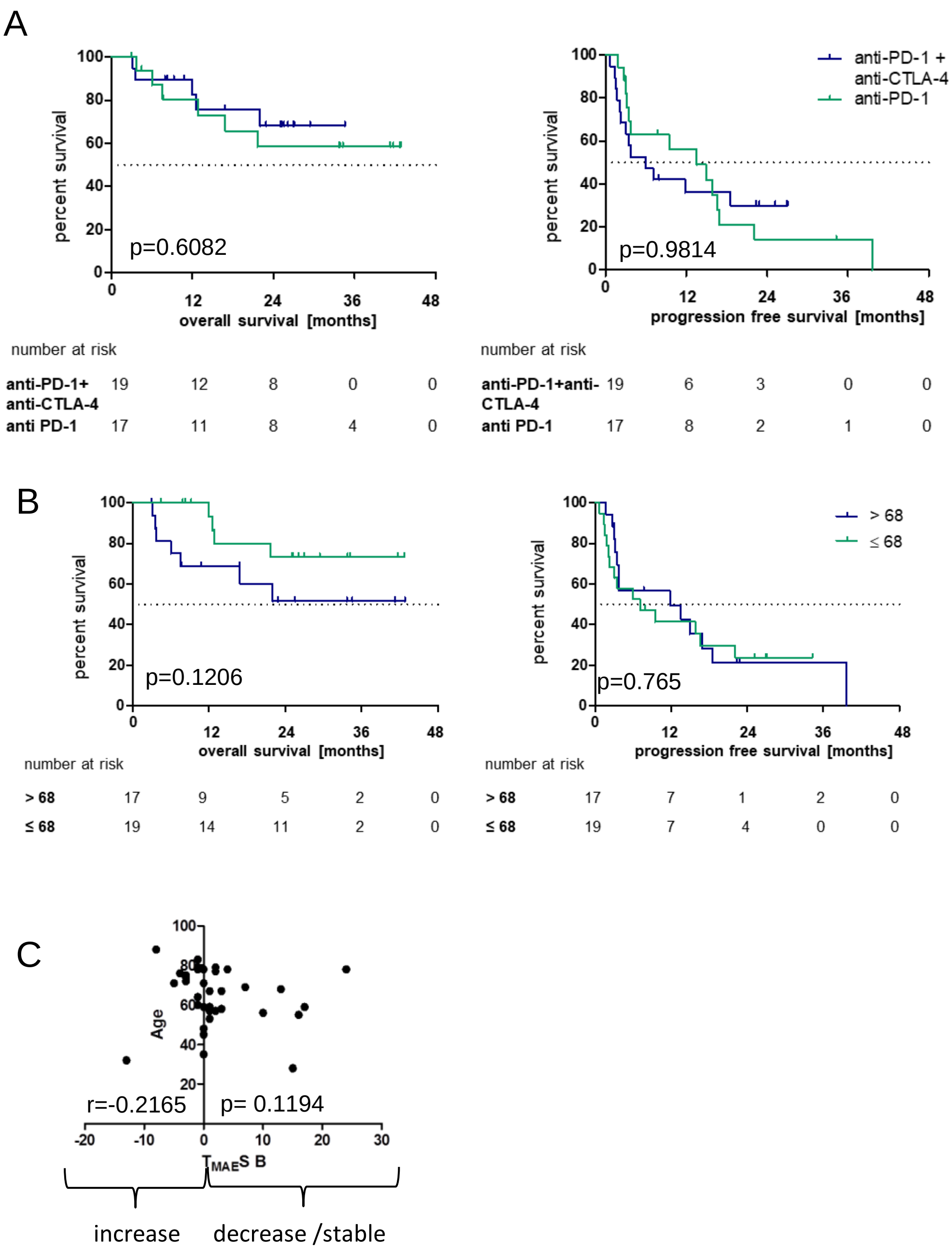

Supplementary Figure 8: Investigation of a potential impact of the applied therapies (A) or age (dichotomization after the median) (B) on patients' OS (left panel) and PFS (right panel). Analysis of a potential correlation between age and T_{MAE}S B using non-parametric Spearman correlation statistics (C).

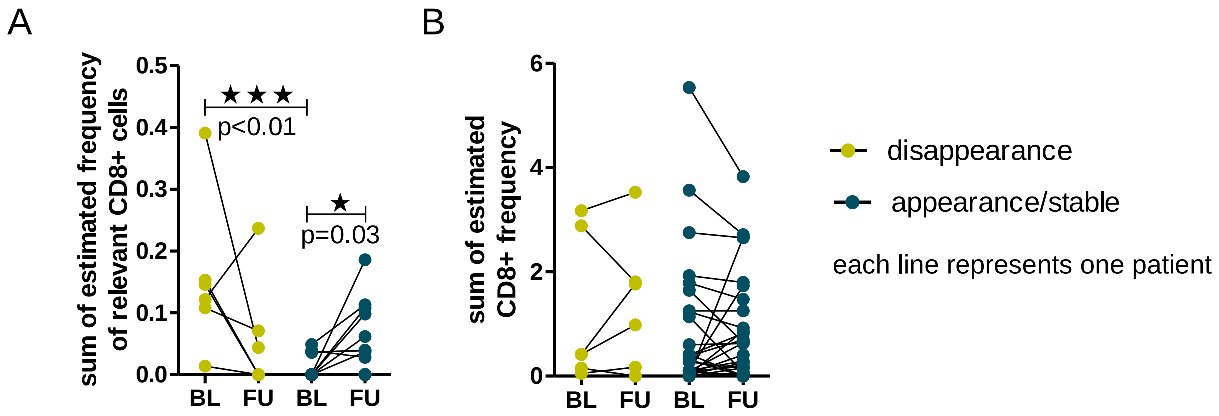

Supplementary Figure 9: Visualization of the sum of estimated frequencies of MAE-specific T cell populations relative to all CD8+ T cells per patient on the basis of the model described in Figure 4 C. Patients were grouped in those with a disappearance (yellow) or those with an appearance/stable T cell population (blue) of at least one of the three most relevant T cell epitopes (TAG-1 SLG, Telomerase RLF and TRP2 SVY MAE)(A).Sum of estimated frequencies of all detected MAE-specific T cell populations (B).

Supplementary Table 1: Overview of the abundance of the MAE-specific CD8+ T cells in samples before and under PD-1 ICB.

**Display Name**	**Antigen Name**	**Epitope sequence**	**BL**	**FU**	**appearance**	**disappearance**	**stable**
707-AP RVA	707-AP	RVAALARDAP	2	3	3	2	0
alpha-actinin-4 FIA	alpha-actinin-4	FIASNGVKLV	1	0	0	1	0
ATIC RLD	ATIC (AICRT)	RLDFNLIRV	0	1	1	0	0
BA46 GLQ	BA46(MFGE8)	GLQHWVPEL	1	0	0	1	0
BAP31 KLD	BAP31	KLDVGNAEV	0	1	1	0	0
Bcl-2 WLS	Bcl-2	WLSLKTLLSL	2	3	2	1	1
Bcl-xL YLN	Bcl-xL	YLNDHLEPWI	0	2	2	0	0
B-RAF LATE	B-RAF	LATEKSRWS	1	2	1	0	1
P-cadherin FII	P-cadherin	FIIENLKAA	4	1	0	3	1
CDCA1/NUF2 KLA	CDCA1/NUF2	KLATAQFKI	1	1	1	1	0
CDK4 ACD	CDK4	ACDPHSGHFV	0	4	4	0	0
CDKN1A FAW	CDKN1A	FAWERVRGL	0	1	1	0	0
CDKN1A GLG	CDKN1A	GLGLPKLYL	7	10	7	4	3
CDKN1A LMA	CDKN1A	LMAGCIQEA	0	2	2	0	0
CLP NLV	CLP (coactosin-like protein)	NLVRDDGSAV	1	2	2	1	0
CLP RLF	CLP (coactosin-like protein)	RLFAFVRFT	2	3	3	2	0
c-MET YVD	c-MET	YVDPVITSI	0	1	1	0	0
CML28 ALV	CML28 (EXOSC5)	ALVDAGVPM	1	0	0	1	0
COA-1 RLL	COA-1 (UBXN11)	RLLASLQDL	1	2	2	1	0
CPSF KVH	CPSF	KVHPVIWSL	0	1	1	0	0
Cyclin B1 AKY	cyclin B1	AKYLMELTM	1	1	1	1	0
Cyclin B1 ILI	cyclin B1	ILIDWLVQV	1	3	2	0	1
Cyclin D1 LLG	cyclin D1	LLGATCMFV	1	2	2	1	0
Cyclophilin B KLK	cyclophilin B (Cyp-B)	KLKHYGPGWV	0	1	1	0	0
DAM-6 FLW	DAM-6, -10	FLWGPRAYA	0	2	2	0	0
EphA2 IMN	EphA2	IMNDMPIYM	0	2	2	0	0
EphA2 VLL	EphA2	VLLLVLAGV	3	5	3	1	2
EZH2 FMV	EZH2	FMVEDETVL	0	1	1	0	0
GnTV VLPV	GnTV	VLPDVFIRCV	3	2	2	3	0
gp100 AML	gp100 / Pmel17	AMLGTHTMEV	0	1	1	0	0
gp100 ITD	gp100 / Pmel17	ITDQVPFSV	1	2	1	0	1
gp100 KTW	gp100 / Pmel17	KTWGQYWQV	1	2	1	0	1
gp100 LLD	gp100 / Pmel17	LLDGTATLRL	4	4	2	2	2
gp100 MLG	gp100 / Pmel17	MLGTHTMEV	0	1	1	0	0
gp100 RLM	gp100 / Pmel17	RLMKQDFSV	0	1	1	0	0
gp100 RLP	gp100 / Pmel17	RLPRIFCSC	1	2	2	1	0
gp100 SLA	gp100 / Pmel17	SLADTNSLAV	0	1	1	0	0
gp100 YLE	gp100 / Pmel17	YLEPGPVTA	2	1	1	2	0
HERV-K-MEL MLA	HERV-K-MEL	MLAVISCAV	1	2	1	0	1
hsp70 LLD	hsp70	LLDVAPLSL	0	1	1	0	0
hsp70 LLL	hsp70	LLLLDVAPL	0	1	1	0	0
IDO1 ALL	IDO1	ALLEIASCL	0	2	2	0	0
IMP-3 NLS	IMP-3	NLSSAEVVV	1	0	0	1	0
KIF20A AQP	KIF20A	AQPDTAPLPV	3	6	3	0	3
KIF20A LLS	KIF20A	LLSDDDVVV	1	3	2	0	1
LAGE-1 MLM	LAGE-1	MLMAQEALAFL	3	4	3	2	1
Livin RLA	Livin (ML-IAP)	RLASFYDWLP	3	3	2	2	1
Livin SLG	Livin (ML-IAP)	SLGSPVLGL	3	4	3	2	1
M2BP RID	M2BP	RIDITLSSV	1	1	1	1	0
MAGE-A1 KVL	MAGE-A1	KVLEYVIKV	4	4	3	3	1
MAGE-A1 YLE	MAGE-A1	YLEYRQVPV	4	4	3	3	1
MAGE-A10 GLY	MAGE-A10	GLYDGMEHL	1	1	1	1	0
MAGE-A10 SLL	MAGE-A10	SLLKFLAKV	7	5	1	3	4
MAGE-A12 FLW	MAGE-A12	FLWGPRALV	1	2	2	1	0
MAGE-A2 KMV	MAGE-A2	KMVELVHFL	2	4	4	2	0

MAGE-A2 LVH	MAGE-A2	LVHFLLLKY	13	13	1	1	12
MAGE-A2 LVQ	MAGE-A2	LVQENYLEY	1	1	1	1	0
MAGE-A2 YLQ	MAGE-A2	YLQLVFGIEV	4	4	3	3	1
MAGE-A3 KVA	MAGE-A3	KVAELVHFL	0	5	5	0	0
MAGE-A4 GVY	MAGE-A4	GVYDGREHTV	1	2	1	0	1
MAGE-A8 KVA	MAGE-A8	KVAELVRFL	2	0	0	2	0
MAGE-A9 ALS	MAGE-A9	ALSVMGVYV	0	2	2	0	0
MAGE-C2 ALK	MAGE-C2	ALKDVEERV	1	2	2	1	0
MAGE-C2 KVL	MAGE-C2	KVLEFLAKL	8	6	3	5	3
MAGE-C2 LLF	MAGE-C2	LLFGLALIEV	4	4	1	1	3
MAGE-C2 TLD	MAGE-C2	TLDEKVAELV	1	1	1	1	0
MAGE-C2 VIW	MAGE-C2	VIWEVLNAV	0	1	1	0	0
MC1R TIL	MC1R	TILLGIFFL	4	6	3	1	3
Melan-A AAG	Melan-A / MART-1	AAGIGILTV	1	1	0	0	1
Melan-A EAA	Melan-A / MART-1	EAAGIGILTV	3	4	2	1	2
Melan-A ELA	Melan-A / MART-1	ELAGIGILTV	7	7	2	2	5
Melan-A ILT	Melan-A / MART-1	ILTVILGVL	2	6	5	1	1
Meloe-1 TLN	Meloe-1	TLNDECWPA	1	0	0	1	0
Meloe-2 RLP	Meloe-2	RLPPKPPLA	1	1	1	1	0
MG50 CMH	MG50	CMHLLLEAV	1	3	3	1	0
MG50 LLL	MG50	LLLEAVPAV	0	1	1	0	0
MG50 RLG	MG50	RLGPTLMCL	3	5	3	1	2
MG50 TLK	MG50	TLKCDCEIL	3	4	3	2	1
NY-ESO-1 QLS	NY-ESO-1 / LAGE-2	QLSLLMWIT	3	2	2	3	0
NY-ESO-1 SLL	NY-ESO-1 / LAGE-2	SLLMWITQC	3	2	0	1	2
P Polypeptide IML	P Polypeptide	IMLCLIAAV	1	1	1	1	0
p53 KLC	p53	KLCPVQLWV	0	2	2	0	0
p53 KTC	p53	KTCPVQLWV	3	5	3	1	2
p53 LLG	p53	LLGRNSFEV	2	0	0	2	0
p53 RMP	p53	RMPEAAPPV	4	5	5	4	0
p53 SMP	p53	SMPPPGTRV	2	3	3	2	0
p53 VVP	p53	VVPCEPPEV	1	1	1	1	0
p53 YLG	p53	YLGSYGFRL	1	2	2	1	0
PGK1 IIG	PGK1	IIGGGMAFT	1	0	0	1	0
PRDX5 LLL	PRDX5	LLLDDLLVSI	2	5	4	1	1
RAB38 VLH	RAB38 / NY-MEL-1	VLHWDPETV	0	2	2	0	0
RAGE-1 LKL	RAGE-1	LKLSGVVRL	0	4	4	0	0
RAGE-1 PLP	RAGE-1	PLPPARNGGL	0	1	1	0	0
SART-3 RLA	SART-3	RLAEYQAYI	3	3	1	1	2
Secernin 1 KMD	secernin 1	KMDAEHPEL	3	2	2	3	0
SSX-2 KAS	SSX-2	KASEKIFYV	2	2	0	0	2
SSX-2 RLQ	SSX-2	RLQGISPKI	3	0	0	3	0
STAT1-alpha/ß KLQ	STAT1-alpha/ß	KLQELNYNL	4	6	3	1	3
STEAP1 FLY	STEAP1	FLYTLLREV	2	4	4	2	0
STEAP1 LLL	STEAP1	LLLGTIHAL	1	0	0	1	0
STEAP1 MIA	STEAP1	MIAVFLPIV	6	5	3	4	2
Survivin LML	Survivin	LMLGEFLKL	0	3	3	0	0
Survivin QMF	Survivin	QMFFCFKEL	1	0	0	1	0
TAG-1 SLG	TAG-1	SLGWLFLLL	4	5	3	2	2
Telomerase ILA	Telomerase	ILAKFLHWL	0	4	4	0	0
Telomerase RLFF	Telomerase	RLFFYRKSV	7	6	2	3	4
Topoisomerase II FLY	Topoisomerase II	FLYDDNQRV	1	0	0	1	0
TRAG-3 ILL	TRAG-3	ILLRDAGLV	2	4	3	1	1
TRP-2 FVW	TRP-2	FVWLHYYSV	5	4	2	3	2
TRP-2 SVY	TRP-2	SVYDFFVWL	3	3	2	2	1
TRP-2 TLD	TRP-2	TLDSQVMSL	0	1	1	0	0
TRP-2 VYD	TRP-2	VYDFFVWLHY	2	1	0	1	1
TRP2-6b ATT	TRP2-6b	ATTNILEHY	1	0	0	1	0
Tyrosinase CLL	tyrosinase	CLLWSFQTSA	3	1	1	3	0
Tyrosinase MLL	tyrosinase	MLLAVLYCL	2	3	3	2	0

Tyrosinase YMD	tyrosinase	YMDGTMSQV	0	2	2	0	0
XBP-1 LLS	XBP-1	LLSGQPASA	1	0	0	1	0
adipophilin SVA	adipophilin	SVASTITGV	-	-	-	-	-
ATIC (AICRT) MVY	ATIC (AICRT)	MVYDLYKTL	-	-	-	-	-
BA46 (MFGE8) NLF	BA46 (MFGE8)	NLFETPVEA	-	-	-	-	-
Bcl-2 PLF	Bcl-2	PLFDFSWLSL	-	-	-	-	-
BING-4 CQW	BING-4	CQWGRLWQL	-	-	-	-	-
B-RAF LAT	B-RAF	LATEKSRWSG	-	-	-	-	-
P-cadherin FIL	Cadherin 3/P-cadherin	FILPVLGAV	-	-	-	-	-
CDCA1 YMM	CDCA1/NUF2	YMMPVNSEV	-	-	-	-	-
CLP VVQ	CLP (coactosin-like protein)	VVQNFAKEFV	-	-	-	-	-
COA-1FMT	COA-1 (UBXN11)	FMTRKLWDL	-	-	-	-	-
CPSF LML	CPSF	LMLQNALTTM	-	-	-	-	-
Cyclin I LL	Cyclin I	LLDRFLATV	-	-	-	-	-
cyclin B1 AGY	cyclin B1	AGYLMELCC	-	-	-	-	-
cyclophilin B VLE	cyclophilin B (Cyp-B)	VLEGMEVV	-	-	-	-	-
CYP1B1 WLQ	CYP1B1	WLQYFPNPV	-	-	-	-	-
EphA2 TLA	EphA2	TLADFDPRV	-	-	-	-	-
EphA2 VLA	EphA2	VLAGVGFFI	-	-	-	-	-
EZH2 FIN	EZH2	FINDEIFVEL	-	-	-	-	-
GnTV VLP	GnTV	VLPDVFIRC	-	-	-	-	-
gp100 IMD	gp100 / Pmel17	IMDQVPFSV	-	-	-	-	-
gp100 VLY	gp100 / Pmel17	VLYRYGSFSV	-	-	-	-	-
IMP-3 RLL	IMP-3	RLLVPTQFV	-	-	-	-	-
KIF20A CIA	KIF20A	CIAEQYHTV	-	-	-	-	-
Livin QLC	Livin (ML-IAP)	QLCPICRAPV	-	-	-	-	-
MAGE-A3 LVF	MAGE-A3	LVFGIELMEV	-	-	-	-	-
MAGE-A8 GLM	MAGE-A8	GLMDVQIPT	-	-	-	-	-
MAGE-C2 FLA	MAGE-C2	FLAKLNNTV	-	-	-	-	-
Meloe-2 RCP	Meloe-2	RCPPKPPLA	-	-	-	-	-
MG50 VLS	MG50	VLSVNVPDV	-	-	-	-	-
MG50 WLP	MG50	WLPKILGEV	-	-	-	-	-
NY-ESO-1 SLA	NY-ESO-1 / LAGE-2	SLLMWITQA	-	-	-	-	-
NY-ESO-1 SLL	NY-ESO-1 / LAGE-2	SLLMWITQCFL	-	-	-	-	-
p53 GLA	p53	GLAPPQHLIRV	-	-	-	-	-
p53 LLP	p53	LLPENNVLSPV	-	-	-	-	-
p53 VVP	p53	VVPCEPPEV	-	-	-	-	-
PRAME ALY	PRAME	ALYVDSLFFL	-	-	-	-	-
PRAME SLL	PRAME	SLLQHLIGL	-	-	-	-	-
PRAME SLY	PRAME	SLYSFPEPEA	-	-	-	-	-
PRAME VLD	PRAME	VLDGLDVLL	-	-	-	-	-
PRDX5 AMA	PRDX5	AMAPIKVRL	-	-	-	-	-
Rep. protein A YLM	Replication protein A	YLMDTSGKV	-	-	-	-	-
SART-3 LLQ	SART-3	LLQAEAPRL	-	-	-	-	-
SOX10 AWI	SOX10	AWISKPPGV	-	-	-	-	-
SOX10 SAW	SOX10	SAWISKPPGV	-	-	-	-	-
Survivin ELT	Survivin	ELTLGEFLKL	-	-	-	-	-
Survivin TLP	Survivin	TLPPAWQPFL	-	-	-	-	-
Telomerase RLV	Telomerase	RLVDDFLLV	-	-	-	-	-
Telomerase LLT	Telomerase	LLTSRLRFI	-	-	-	-	-
TRP-2 SLD	TRP-2	SLDDYNHLV	-	-	-	-	-
TYMS LMA	TYMS	LMALPPCHAL	-	-	-	-	-

Supplementary Table 2: Antibody panels that were used to stain multimer+ CD8+ T cells.

marker	***fluorophore***	***clone***	***vendor***	***cat***
CD8	BV480	RPA-T8	BD	566121
CD8	BV510	RPA-T8	BD	563256
CD4	FITC	SK3	BD	345768
CD14	FITC	MΦP9	BD	345784
CD16	FITC	NKP15	BD	335035
CD19	FITC	4G7	BD	345776
CD40	FITC	LOB7/6	BioRad	MCA1590F

Supplementary Table 3: Antibody panels that were used to stain CD8+, CD4+ T cells, Tregs and checkpoint molecules.

marker	***fluorophore***	***clone***	***vendor***	***cat***
CD25	PE	M-A251	BD	555432
CD127	BV510	AO19D5	Biolegend	351322
CD8	APC-fire	SK1	Biolegend	344746
CD8	APC-Cy7	SK1	Biolegend	344714
CD3	A700	UCHT1	Biolegend	300424
CD4	PerCP	SK3	BD	345770
Lag-3	BV421	11C3C65	Biolegend	369314
PD-1	BV711	EH12.2H7	Biolegend	329928
TIM3	BB515	7D3	BD	565569
FoxP3	A647	259DC7	BD	560045
Isotype	BV421	MOPC-21	Biolegend	400158
Isotype	BV711	MOPC-22	Biolegend	400168
Isotype	BB515	X40	BD	564416

Supplementary Table 4: Antibody panels that were used to identify MDSC and classical monocytes.

marker	***fluorophore***	***clone***	***vendor***	***cat***
CD3	BV605	OKT3	Biolegend	317322
CD19	BV605	HIB19	Biolegend	302244
CD16	PacificBlue	3G8	Biolegend	302032
CD11b	APC-fire	ICRF44	Biolegend	301352
CD14	PE-Cy7	M5E2	Biolegend	301814
CD33	FITC	P67.6	Biolegend	366620
HLA-DR	PerCP-Cy5.5	L243	BD	339216
CD56	BV711	HCD56	Biolegend	318336
CD56	BV605	HCD56	Biolegend	318334

Supplementary Table 5: Estimated frequencies of the detected MAE-specific CD8+ T cell populations in each patient at BL and FU.

Patient	Name	baseline	follow-up
**1**	MAGE-A10 SLL	0,061%	0,113%
	MAGE-A1 YLE	0.000%	0.025%
	Melan-A ILT	0.000%	0.019%
	CDK4 ACD	0.000%	0.019%
	MAGE-A1 KVL	0.000%	0.017%
	CDKN1A GLG	0.000%	0.017%
	KIF20A AQP	0.000%	0.016%
	p53 KLC	0.000%	0.015%
	DAM-6 FLW	0.000%	0.015%
	p53 SMP	0.000%	0.013%
	gp100 RLP	0.000%	0.011%
	MG50 TLK	0.000%	0.011%
	MAGE-A2 LVH	0.045%	0.000%
**2**	MAGE-A10 SLL	0.282%	0.000%
	MAGE-A1 YLE	0.059%	0.000%
	CDKN1A GLG	0.045%	0.000%
	MG50 TLK	0.029%	0.000%
**3**	Bcl-2 WLS	0.000%	0.346%
	MAGE-A2 LVH	0.009%	0.062%
	MAGE-A10 GLY	0.000%	0.057%
	MAGE-A10 SLL	0.020%	0.053%
	RAB38 VLH	0.000%	0.021%
	COA-1 RLL	0.000%	0.012%
	MAGE-A1 YLE	0.000%	0.012%
	EphA2 VLL	0.018%	0.010%
	Bcl-xL YLN	0.000%	0.010%
	CDK4 ACD	0.000%	0.008%
	CDKN1A GLG	0.000%	0.008%
	MAGE-A1 KVL	0.000%	0.008%
	RAGE-1 LKL	0.000%	0.007%
	KIF20A AQP	0.000%	0.007%
	p53 SMP	0.000%	0.006%
	MG50 TLK	0.000%	0.006%
	gp100 SLA	0.000%	0.005%
	Cyclin D1 LLG	0.042%	0.000%
5	gp100 KTW	0.043%	0.554%
	Melan-A ELA	0.000%	0.116%
	ATIC RLD	0.000%	0.098%
	MG50 CMH	0.000%	0.097%
7	MAGE-A2 LVH	1.054%	0.068%
	NY-ESO-1 QLS	0.088%	0.000%
8	gp100 LLD	0.000%	0.564%
	HERV-K-MEL MLA	0.000%	0.086%
	MAGE-A2 YLQ	0.499%	0.000%
	Melan-A ELA	0.349%	0.000%
	CDKN1A GLG	0.310%	0.000%
	p53 YLG	0.298%	0.000%
	MAGE-C2 KVL	0.192%	0.000%
4	MAGE-A10 SLL	0.119%	0.224%
	MAGE-A2 LVH	0.141%	0.118%
	MAGE-A1 YLE	0.025%	0.040%
	CDK4 ACD	0.000%	0.027%
	CDKN1A GLG	0.019%	0.026%
	KIF20A AQP	0.018%	0.026%
	Melan-A ILT	0.000%	0.023%
	MAGE-A1 KVL	0.019%	0.023%
	DAM-6 FLW	0.000%	0.021%
	p53 KLC	0.000%	0.020%
	p53 SMP	0.000%	0.018%
	MG50 TLK	0.012%	0.017%
	CDKN1A LMA	0.000%	0.015%
	M2BP RID	0.000%	0.014%
	gp100 RLP	0.000%	0.013%
	MAGE-A4 GVY	0.000%	0.010%
	PRDX5 LLL	0.000%	0.010%
	gp100 RLM	0.000%	0.009%
	TRP-2 VYD	0.033%	0.008%
	MG50 LLL	0.000%	0.008%
	c-MET YVD	0.000%	0.008%
	EphA2 IMN	0.000%	0.007%
	MAGE-A2 LVQ	0.000%	0.006%
	Cyclin B1 ILI	0.000%	0.006%
6	STEAP1 MIA	0.055%	0.253%
	Livin RLA	0.000%	0.165%
	CLP RLF	0.000%	0.144%
	SSX-2 KAS	0.040%	0.113%
	Melan-A ELA	0.000%	0.084%
	P Polypeptide IML	0.000%	0.067%
	TRAG-3 ILL	0.000%	0.060%
	MAGE-C2 KVL	0.066%	0.059%
	KIF20A AQP	0.000%	0.050%
	gp100 KTW	0.000%	0.048%
	EphA2 IMN	0.000%	0.045%
	707-AP RVA	0.000%	0.043%
	STAT1-alpha/ß KLQ	0.000%	0.041%
	TRP-2 FVW	0.053%	0.039%
	PRDX5 LLL	0.000%	0.024%
	CDKN1A GLG	0.000%	0.022%
	gp100 LLD	0.353%	0.000%
	MAGE-A1 KVL	0.143%	0.000%
	MAGE-A2 YLQ	0.100%	0.000%
	MAGE-A1 YLE	0.096%	0.000%
	NY-ESO-1 QLS	0.081%	0.000%
	PRDX5 LLL	0.064%	0.000%
	P-cadherin FII	0.062%	0.000%
	GnTV VLPV	0.041%	0.000%
	STAT1-alpha/ß KLQ	0.034%	0.000%
	p53 RMP	0.034%	0.000%
	SART-3 RLA	0.028%	0.000%

	gp100 AML	0.000%	1.295%		NY-ESO-1 QLS	0.000%	0.247%
	Melan-A ELA	0.577%	0.197%		Survivin LML	0.000%	0.217%
	STAT1-alpha/ß KLQ	0.000%	0.113%		MAGE-C2 ALK	0.000%	0.144%
	Livin SLG	0.267%	0.109%		Melan-A ELA	0.136%	0.137%
	Tyrosinase MLL	0.000%	0.072%		CLP NLV	0.000%	0.134%
	CDKN1A GLG	0.000%	0.029%		MG50 CMH	0.000%	0.125%
	MAGE-A2 KMV	0.337%	0.000%		Telomerase ILA	0.000%	0.123%
	p53 RMP	0.280%	0.000%		STEAP1 FLY	0.000%	0.114%
	MAGE-A10 SLL	0.273%	0.000%		Telomerase RLF	0.049%	0.113%
9	STEAP1 MIA	0.196%	0.000%		MAGE-C2 VIW	0.000%	0.113%
	MAGE-C2 KVL	0.195%	0.000%		MAGE-A1 YLE	0.291%	0.000%
	MAGE-A1 KVL	0.171%	0.000%		Livin RLA	0.180%	0.000%
	gp100 YLE	0.128%	0.000%		TRP-2 VYD	0.112%	0.000%
	Tyrosinase CLL	0.127%	0.000%		Cyclin D1 LLG	0.109%	0.000%
	Melan-A EAA	0.098%	0.000%		MG50 RLG	0.108%	0.000%
	TAG-1 SLG	0.077%	0.000%	10	LAGE-1 MLM	0.102%	0.000%
	Telomerase RLF	0.076%	0.000%		MAGE-A2 KMV	0.080%	0.000%
	gp100 LLD	0.075%	0.000%		STAT1-alpha/ß KLQ	0.070%	0.000%
	Melan-A ELA	0.000%	1.095%		MAGE-A10 GLY	0.065%	0.000%
	MAGE-A12 FLW	0.000%	0.960%		MAGE-A2 YLQ	0.065%	0.000%
	MG50 RLG	0.000%	0.593%		MAGE-C2 KVL	0.064%	0.000%
	LAGE-1 MLM	0.000%	0.318%		Livin SLG	0.060%	0.000%
	MAGE-C2 LLF	0.049%	0.288%		Meloe-1 TLN	0.059%	0.000%
	Melan-A EAA	0.185%	0.149%		TRP-2 FVW	0.048%	0.000%
	KIF20A AQP	0.021%	0.087%		707-AP RVA	0.041%	0.000%
	SSX-2 KAS	0.141%	0.069%		STEAP1 MIA	0.041%	0.000%
	MAGE-A3 KVA	0.000%	0.038%		P-cadherin FII	0.035%	0.000%
	NY-ESO-1 QLS	0.000%	0.037%		CLP RLF	0.027%	0.000%
	MAGE-A2 KMV	0.000%	0.034%		p53 KTC	0.023%	0.000%
	MAGE-A2 YLQ	0.000%	0.028%		SSX-2 RLQ	0.013%	0.000%
	TAG-1 SLG	0.000%	0.023%		CDKN1A GLG	0.011%	0.000%
	Tyrosinase YMD	0.000%	0.022%	21	gp100 YLE	0.101%	0.000%
	Tyrosinase MLL	0.000%	0.020%	22	-	-	-
11	gp100 LLD	3.334%	0.017%		Livin SLG	0.000%	0.746%
	Telomerase RLFF	0.000%	0.016%		p53 RMP	0.000%	0.363%
	TRP-2 FVW	0.000%	0.016%		707-AP RVA	0.000%	0.348%
	GnTV VLPV	0.000%	0.014%		MAGE-C2 KVL	0.000%	0.253%
	PRDX5 LLL	0.000%	0.010%		TAG-1 SLG	0.000%	0.186%
	Melan-A ELA	1.516%	0.000%		Livin RLA	0.000%	0.183%
	p53 RMP	0.094%	0.000%		STEAP1 MIA	0.000%	0.129%
	Tyrosinase CLL	0.038%	0.000%	23	MAGE-A2 YLQ	0.000%	0.119%
	Telomerase RLF	0.037%	0.000%		MAGE-A3 KVA	0.000%	0.059%
	STEAP1 MIA	0.026%	0.000%		MAGE-A1 YLE	0.000%	0.052%
	CLP NLV	0.026%	0.000%		p53 KTC	0.000%	0.045%
	MAGE-C2 KVL	0.021%	0.000%		CDKN1A GLG	0.000%	0.044%
	Livin RLA	0.018%	0.000%		B-RAF LATE	0.000%	0.043%
	Secernin 1 KMD	0.018%	0.000%		Meloe-2 RLP	0.000%	0.041%
	PGK1 IIG	0.016%	0.000%		CDKN1A FAW	0.000%	0.033%
	XBP-1 LLS	0.004%	0.000%		CLP NLV	0.000%	0.022%

12	gp100 LLD	0.052%	2.242%
	MAGE-C2 KVL	0.098%	0.436%
	KIF20A LLS	0.000%	0.086%
	P-cadherin FII	0.256%	0.073%
	TAG-1 SLG	0.079%	0.071%
	MAGE-A2 YLQ	0.000%	0.064%
	MAGE-A3 KVA	0.000%	0.060%
	p53 RMP	0.000%	0.056%
	MAGE-A1 KVL	0.000%	0.053%
	LAGE-1 MLM	0.000%	0.048%
	p53 KTC	0.026%	0.043%
	707-AP RVA	0.000%	0.041%
	gp100 MLG	0.000%	0.038%
	Cyclin D1 LLG	0.000%	0.034%
	Livin RLA	0.072%	0.033%
	STEAP1 MIA	0.128%	0.032%
	MAGE-A2 KMV	0.000%	0.028%
	Melan-A EAA	0.000%	0.022%
	MG50 RLG	0.023%	0.020%
	SART-3 RLA	0.106%	0.019%
	STAT1-alpha/ß KLQ	0.020%	0.017%
	CDKN1A GLG	0.072%	0.013%
	MAGE-A10 SLL	0.955%	0.000%
	Cyclin D1 LLG	0.577%	0.000%
	TRP-2 FVW	0.207%	0.000%
	Secernin 1 KMD	0.110%	0.000%
	Livin SLG	0.085%	0.000%
	MAGE-A2 LVQ	0.068%	0.000%
	Tyrosinase MLL	0.061%	0.000%
	P Polypeptide IML	0.053%	0.000%
	KIF20A LLS	0.049%	0.000%
	Telomerase RLF	0.029%	0.000%
	Tyrosinase CLL	0.026%	0.000%
	SSX-2 RLQ	0.015%	0.000%
20	EZH2 FMV	0.000%	0.180%
	MAGE-C2 KVL	0.000%	0.108%
	Cyclophilin B KLK	0.000%	0.102%
	Telomerase RLF	0.000%	0.098%
	p53 YLG	0.000%	0.085%
	hsp70 LLD	0.000%	0.078%
	CDKN1A LMA	0.000%	0.066%
	gp100 YLE	0.000%	0.059%
	Survivin LML	0.000%	0.054%
	CDK4 ACD	0.000%	0.050%
	CDCA1/NUF2 KLA	0.000%	0.042%
	MG50 TLK	0.316%	0.000%
	TRP2-6b ATT	0.205%	0.000%
	p53 SMP	0.140%	0.000%
	p53 RMP	0.110%	0.000%
	STEAP1 LLL	0.101%	0.000%
	MAGE-A12 FLW	0.086%	0.000%
	MAGE-C2 TLD	0.082%	0.000%
	alpha-actinin-4 FIA	0.073%	0.000%
	IMP-3 NLS	0.072%	0.000%
	CLP RLF	0.051%	0.000%

13	MAGE-A1 KVL	0.042%	0.933%
	p53 KTC	0.000%	0.265%
	TRP-2 FVW	0.085%	0.224%
	Secernin 1 KMD	0.000%	0.202%
	LAGE-1 MLM	0.282%	0.194%
	p53 RMP	0.000%	0.098%
	MAGE-C2 KVL	0.220%	0.092%
	Tyrosinase MLL	0.000%	0.073%
	STAT1-alpha/ß KLQ	0.000%	0.064%
	MAGE-A2 KMV	0.000%	0.062%
	STEAP1 MIA	0.000%	0.060%
	BAP31 KLD	0.000%	0.055%
	MG50 TLK	0.000%	0.054%
	Livin SLG	0.000%	0.053%
	Telomerase ILA	0.000%	0.052%
	SART-3 RLA	0.032%	0.044%
	Telomerase RLF	0.036%	0.040%
	Tyrosinase CLL	0.000%	0.035%
	MG50 RLG	0.000%	0.031%
	TRAG-3 ILL	0.024%	0.023%
	707-AP RVA	0.949%	0.000%
	p53 VVP	0.817%	0.000%
	GnTV VLPV	0.092%	0.000%
	MAGE-C2 ALK	0.073%	0.000%
	p53 SMP	0.055%	0.000%
	MAGE-A8 KVA	0.039%	0.002%
15	CDKN1A GLG	0.000%	0.257%
	MAGE-C2 KVL	0.000%	0.127%
	p53 KTC	0.000%	0.127%
	Livin SLG	0.000%	0.110%
	CLP RLF	0.000%	0.102%
	STEAP1 MIA	0.000%	0.096%
	LAGE-1 MLM	0.000%	0.063%
	MAGE-A3 KVA	0.000%	0.063%
	MAGE-C2 ALK	0.000%	0.057%
	p53 VVP	0.000%	0.057%
	p53 YLG	0.000%	0.053%
	Secernin 1 KMD	0.000%	0.051%
	MAGE-A2 KMV	0.000%	0.050%
	Cyclin D1 LLG	0.000%	0.048%
	MAGE-A10 SLL	0.037%	0.047%
	STAT1-alpha/ß KLQ	0.000%	0.043%
	Tyrosinase YMD	0.000%	0.042%
	MAGE-A12 FLW	0.000%	0.041%
	GnTV VLPV	0.000%	0.040%
	p53 RMP	0.000%	0.038%
	KIF20A AQP	0.037%	0.037%
	Telomerase RLF	0.000%	0.037%
	Telomerase ILA	0.000%	0.035%
	KIF20A LLS	0.000%	0.031%
	MG50 RLG	0.038%	0.031%
	SART-3 RLA	0.000%	0.027%
	PRDX5 LLL	0.000%	0.015%

14	MAGE-A3 KVA	0.000%	1.515%
	gp100 LLD	0.000%	0.673%
	Melan-A ELA	0.080%	0.100%
	Meloe-2 RLP	0.092%	0.097%
	IDO1 ALL	0.000%	0.046%
	MAGE-A2 YLQ	0.099%	0.045%
	MG50 RLG	0.000%	0.036%
	MAGE-C2 LLF	0.053%	0.031%
	HERV-K-MEL MLA	0.078%	0.031%
	MAGE-A4 GVY	0.035%	0.029%
	Telomerase RLF	0.039%	0.028%
	STAT1-alpha/ß KLQ	0.862%	0.018%
	KIF20A LLS	0.000%	0.018%
	Melan-A EAA	0.000%	0.017%
	p53 KTC	0.027%	0.017%
	CDKN1A GLG	0.009%	0.012%
	MAGE-C2 KVL	1.071%	0.000%
	Survivin QMF	0.495%	0.000%
	Secernin 1 KMD	0.213%	0.000%
	Tyrosinase MLL	0.123%	0.000%
	NY-ESO-1 QLS	0.067%	0.000%
	M2BP RID	0.063%	0.000%
	LAGE-1 MLM	0.063%	0.000%
	STEAP1 MIA	0.037%	0.000%
	TRP-2 FVW	0.037%	0.000%
	MAGE-A8 KVA	0.020%	0.000%
	PRDX5 LLL	0.010%	0.000%
16	TAG-1 SLG	0.146%	0.000%
17	Survivin LML	0.000%	0.165%
18	-	-	-
19	TRP-2 TLD	0.000%	0.113%
	CLP RLF	0.000%	0.041%
	GnTV VLPV	0.108%	0.000%
	BA46 GLQ	0.094%	0.000%
	CDCA1/NUF2 KLA	0.071%	0.000%
24	MG50 CMH	0.316%	0.000%
25	CPSF KVH	0.000%	0.048%
26	-	-	-
27	EphA2 VLL	0.000%	0.146%
	MAGE-A2 LVH	0.021%	0.051%
	TRAG-3 ILL	0.000%	0.046%
	SSX-2 RLQ	0.064%	0.000%
	MC1R TIL	0.040%	0.000%
28	MAGE-A2 LVH	0.030%	0.217%
	Melan-A ILT	0.000%	0.124%
	MC1R TIL	0.000%	0.039%
29	MAGE-A2 LVH	0.044%	0.188%
	MC1R TIL	0.000%	0.090%
	TRP-2 SVY	0.016%	0.000%
30	Melan-A ELA	0.358%	0.955%
	Melan-A EAA	0.261%	0.656%
	Melan-A AAG	0.109%	0.256%
	MC1R TIL	0.129%	0.206%
	TRP-2 SVY	0.000%	0.135%
	MAGE-A2 LVH	0.381%	0.051%
	STEAP1 FLY	0.429%	0.000%
	Melan-A ILT	0.128%	0.000%
	CML28 ALV	0.061%	0.000%
	Topoisomerase II FLY	0.030%	0.000%
31	MAGE-A2 LVH	0.000%	0.260%
	MC1R TIL	0.022%	0.073%
	MAGE-A9 ALS	0.000%	0.044%
	Melan-A ELA	0.063%	0.000%
	P-cadherin FII	0.037%	0.000%
32	MAGE-A2 LVH	0.016%	0.060%
	MC1R TIL	0.000%	0.041%
	Melan-A ILT	0.000%	0.015%
33	MC1R TIL	0.049%	0.656%
	MAGE-A2 LVH	0.028%	0.613%
	Melan-A ILT	0.067%	0.250%
	STEAP1 FLY	0.000%	0.223%
	EphA2 VLL	0.823%	0.000%
	TRAG-3 ILL	0.250%	0.000%
	COA-1 RLL	0.091%	0.000%
	Bcl-2 WLS	0.042%	0.000%

34	Cyclin B1 ILI	0.014%	0.499%
	TAG-1 SLG	0.000%	0.237%
	RAGE-1 LKL	0.000%	0.190%
	STEAP1 FLY	0.000%	0.158%
	NY-ESO-1 SLL	0.043%	0.108%
	Bcl-2 WLS	0.055%	0.098%
	EphA2 VLL	0.017%	0.091%
	gp100 ITD	0.000%	0.086%
	PRDX5 LLL	0.000%	0.084%
	MAGE-A9 ALS	0.000%	0.077%
	B-RAF LATE	0.026%	0.069%
	MAGE-C2 LLF	0.056%	0.046%
	MAGE-A2 LVH	0.022%	0.026%
	TRP-2 SVY	0.122%	0.000%
	Cyclin B1 AKY	0.035%	0.000%
	CDKN1A GLG	0.028%	0.000%
36	hsp70 LLL	0.000%	0.281%
	TRP-2 FVW	0.000%	0.146%
	RAGE-1 LKL	0.000%	0.077%
	TRP-2 SVY	0.000%	0.062%
	EphA2 VLL	0.000%	0.043%
	MAGE-A2 LVH	0.024%	0.022%
	STEAP1 FLY	0.289%	0.000%
	MAGE-C2 LLF	0.154%	0.000%
	NY-ESO-1 SLL	0.134%	0.000%

35	Bcl-2 WLS	0.000%	0.207%
	EphA2 VLL	0.000%	0.166%
	NY-ESO-1 SLL	0.017%	0.106%
	MAGE-A2 LVH	0.002%	0.060%
	CDKN1A GLG	0.000%	0.054%
	TRAG-3 ILL	0.000%	0.052%
	Cyclin B1 ILI	0.000%	0.050%
	RAGE-1 LKL	0.000%	0.040%
	MG50 CMH	0.000%	0.030%
	RAGE-1 PLP	0.000%	0.029%
	TAG-1 SLG	0.032%	0.027%
	Cyclin B1 AKY	0.000%	0.026%
	COA-1 RLL	0.000%	0.024%
	TRP-2 SVY	0.347%	0.016%
	RAB38 VLH	0.000%	0.016%
	STEAP1 FLY	0.000%	0.016%
	MAGE-C2 LLF	0.000%	0.015%
	Bcl-xL YLN	0.000%	0.014%
	Melan-A ILT	0.000%	0.013%
	gp100 ITD	0.009%	0.012%
	p53 RMP	0.000%	0.007%
	MAGE-A10 SLL	0.000%	0.001%
	Telomerase RLF	0.012%	0.000%
	gp100 RLP	0.003%	0.000%

Supplementary Table 6: Identified correlations of MAE-specific CD8+ T cell populations with OS using a trained elastic net approach.

alpha	accuracy training set	accuracy test set	Identified MAE-specific CD8+ T cell populations
1	0.66	0.85	
0.9	0.72	0.85	TAG-1 SLG
0.8	0.65	0.85	
0.7	***0.89***	***0.85***	***P-cadherin FII; MAGE-A10 SLL; STEAP1 FLY; TAG-1 SLG; Telomerase ILA; Telomerase RLFF; TRAG-3 ILL; TRP-2 SVY; Tyrosinase CLL***
0.6	0.79	0.85	TAG-1 SLG; Telomerase RLF; Tyrosinase CLL
0.5	0.96	0.85	Bcl-xL YLN; P-cadherin FII; Cyclin B1 AKY; EphA2 VLL; gp100 RLP; LAGE-1 MLM; MAGE-A10 GLY; MAGE-A10 SLL; MAGE-A9 ALS; Melan-A EAA; Melan-A ELA; MG50 TLK; NY-ESO-1 QLS; RAB38 VLH; STAT1-alpha/β KLQ; STEAP1 FLY; TAG-1 SLG; Telomerase ILA; Telomerase RLF; TRAG-3 ILL; TRP-2 SVY; Tyrosinase CLL
0.4	0.93	0.85	Bcl-xL YLN; P-cadherin FII; EphA2 VLL; LAGE-1 MLM; MAGE-A10 SLL; RAB38 VLH; STEAP1 FLY; TAG-1 SLG; Telomerase ILA; Telomerase RLF; TRAG-3 ILL; TRP-2 SVY; Tyrosinase CLL
0.3	0.96	0.71	ATIC RLD; Bcl-xL YLN; P-cadherin FII; CLP NLV; Cyclin B1 AKY; EphA2 VLL; gp100 AML; gp100 RLP; gp100 YLE; LAGE-1 MLM; MAGE-A10 GLY; MAGE-A10 SLL; MAGE-A9 ALS; MAGE-C2 KVL; MAGE-C2 LLF; Melan-A EAA; Melan-A ELA; MG50 TLK; NY-ESO-1 QLS; RAB38 VLH; SSX-2 RLQ; STAT1-alpha/β KLQ; STEAP1 FLY; Survivin LML; TAG-1 SLG; Telomerase ILA; Telomerase RLF; TRAG-3 ILL; TRP-2 SVY; Tyrosinase CLL; Tyrosinase MLL
0.2	0.65	0.85	
0.1	0.93	0.42	707-AP RVA; ATIC RLD; Bcl-xL YLN; P-cadherin FII; CLP NLV; CML28 ALV; Cyclin B1 AKY; Cyclin B1 ILI; Cyclin D1 LLG; DAM-6 FLW; EphA2 VLL; GnTV VLPV; gp100 AML gp100 ITD; gp100 LLD; gp100 RLP; gp100 SLA; gp100 YLE; HERV-K-MEL MLA; IDO1 ALL; KIF20A LLS; LAGE-1 MLM; Livin SLG; M2BP RID; MAGE-A1 YLE; MAGE-A10 GLY; MAGE-A10 SLL; MAGE-A2 KMV; MAGE-A2 YLQ; MAGE-A3 KVA; MAGE-A8 KVA; MAGE-A9 ALS; MAGE-C2 KVL; MAGE-C2 LLF; MAGE-C2 VIW; MC1R TIL; Melan-A ILT; Melan-A EAA: Melan-A ELA; Meloe-1 TLN; MG50 TLK; NY-ESO-1 QLS; P Polypeptide IML; p53 KLC; p53 RMP; p53 SMP; p53 YLG; RAB38 VLH; RAGE-1 PLP; Secernin 1 KMD; SSX-2 RLQ; STAT1-alpha/β KLQ; STEAP1 FLY; STEAP1 MIA; Survivin LML; Survivin QMF; TAG-1 SLG; Telomerase ILA; Telomerase RLF; Topoisomerase II FLY; TRAG-3 ILL; TRP-2 SVY; TRP-2 VYD; Tyrosinase CLL; Tyrosinase MLL

Supplementary Table 7: Result of the univariate cox regression of the MAE-specific CD8+ T cell populations. that were identified as associated with OS a in trained elastic net approach.

	LRT	HR	Wald	log rank
**TAG-1 SLG**	0.002	0.039	0.001	0.006
**Telomerase RLF**	0.006	0.096	0.002	0.001
**MAGE-A10 SLL**	0.025	0.107	0.010	0.009
**Tyrosinase CLL**	0.063	0.197	0.034	0.045
**TRAG-3 ILL**	0.235	0.215	0.283	0.303
**STEAP1 FLY**	0.243	2.180	0.229	0.235
**TRP-2 SVY**	0.288	0.424	0.272	0.287
**Telomerase ILA**	0.074	4.43E-09	0.999	0.190
**P-cadherin FII**	0.092	81728653.8	0.998	0.217

LRT: likelihood ratio test. HR: hazard ratio
